# Understanding and Preventing Photoluminescence Quenching to Achieve Unity Photoluminescence Quantum Yield in Yb:YLF Nanocrystals

**DOI:** 10.1021/acsami.2c17888

**Published:** 2023-01-06

**Authors:** Jence T. Mulder, Michael S. Meijer, J. Jasper van Blaaderen, Indy du Fossé, Kellie Jenkinson, Sara Bals, Liberato Manna, Arjan J. Houtepen

**Affiliations:** † Optoelectronic Materials Section, Faculty of Applied Sciences, Delft University of Technology, Van der Maasweg 9, 2629HZ Delft, The Netherlands; ‡ Electron Microscopy for Materials Science (EMAT), Department of Physics, University of Antwerp, Groenenborgerlaan 171, 2020 Antwerp, Belgium; § Department of Nanochemistry, Istituto Italiano di Tecnologia (IIT), Via Morego 30, 16163 Genova, Italy

**Keywords:** luminescence, nanocrystals, rare earth ions, optical refrigeration, core/shell, energy transfer, ytterbium

## Abstract

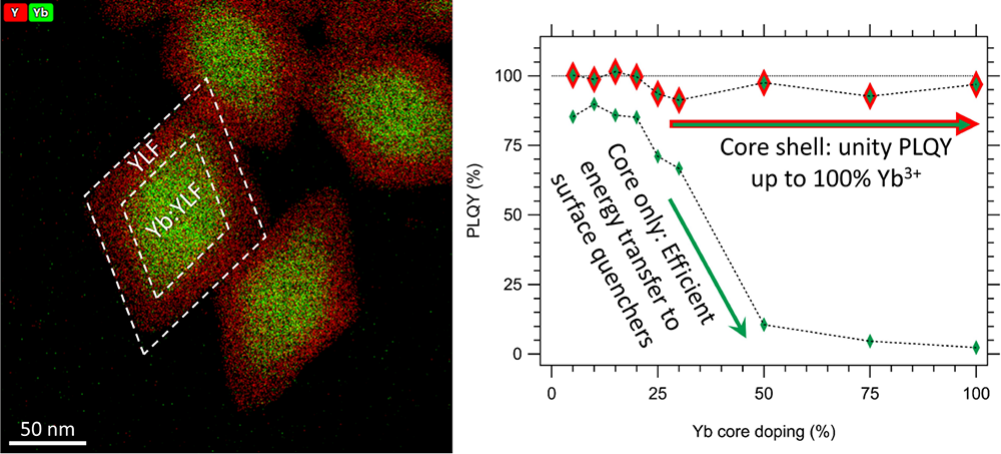

Ytterbium-doped LiYF_4_ (Yb:YLF) is a commonly used material for laser applications, as a photon upconversion medium, and for optical refrigeration. As nanocrystals (NCs), the material is also of interest for biological and physical applications. Unfortunately, as with most phosphors, with the reduction in size comes a large reduction of the photoluminescence quantum yield (PLQY), which is typically associated with an increase in surface-related PL quenching. Here, we report the synthesis of bipyramidal Yb:YLF NCs with a short axis of ∼60 nm. We systematically study and remove all sources of PL quenching in these NCs. By chemically removing all traces of water from the reaction mixture, we obtain NCs that exhibit a near-unity PLQY for an Yb^3+^ concentration below 20%. At higher Yb^3+^ concentrations, efficient concentration quenching occurs. The surface PL quenching is mitigated by growing an undoped YLF shell around the NC core, resulting in near-unity PLQY values even for fully Yb^3+^-based LiYbF_4_ cores. This unambiguously shows that the only remaining quenching sites in core-only Yb:YLF NCs reside on the surface and that concentration quenching is due to energy transfer to the surface. Monte Carlo simulations can reproduce the concentration dependence of the PLQY. Surprisingly, Förster resonance energy transfer does not give satisfactory agreement with the experimental data, whereas nearest-neighbor energy transfer does. This work demonstrates that Yb^3+^-based nanophosphors can be synthesized with a quality close to that of bulk single crystals. The high Yb^3+^ concentration in the LiYbF_4_/LiYF_4_ core/shell nanocrystals increases the weak Yb^3+^ absorption, making these materials highly promising for fundamental studies and increasing their effectiveness in bioapplications and optical refrigeration.

## Introduction

Ytterbium-doped LiYF_4_ (Yb:YLF) is a well-known luminescent material, much used in, *e.g.*, laser applications, for its unity photoluminescence quantum yield (PLQY) that is commonly achieved in bulk single crystals.^1,2^ Co-doped with other rare earth ions, it is broadly investigated for PL upconversion,^3−6^ whereas Yb:YLF itself has also been shown to exhibit optical refrigeration, allowing the remote cooling of samples.^7−10^ For many applications, a small size of the crystals, in the nanometer range, is desired.^11^ This is, for example, the case when miniaturizing the application, as in microleds.^12^ Another important application is in biological experiments,^13^ where nanocrystals (NCs) of <50 nm are required.^14^ Moreover, optical refrigeration using NCs allows the reduction of the internal temperature remotely. This is especially relevant for physical studies of the properties of single nanocrystals at low temperatures.^15^

There are various challenges in using and researching Yb:YLF NCs. First, the unity PLQY that is achieved for bulk Yb:YLF crystals is strongly reduced in NCs, limiting their applicability. There are many potential causes for this poor PLQY (as will be discussed in detail below). The literature suggests that internal quenching via hydroxide (OH^–^) impurities and surface quenching by hydroxyl groups of solvent molecules (−OH) or OH^–^ surface groups are dominant.^16−21^ Second, concentration quenching of the PLQY is almost always observed, leading to the use of low dopant concentrations (≤20%) with concomitant low absorption.^22,23^

Systematic studies that determine which quenching routes are dominant are scarce and focus on NaYF_4_ and upconverting NCs.^24,25^ A major challenge in the study of PL quenching in these materials is that it is notoriously hard to determine the PLQY of Yb:YLF NCs by conventional means, such as making use of reference dyes or calibrated integrating spheres. This is caused by (1) the low absorption of the parity-forbidden ^2^F_7/2_ → ^2^F_5/2_ transition, complicating the quantification of the number of absorbed photons, and (2) the emission wavelength around 1000 nm, where good reference dyes are absent.

This leads to the following research questions that we address in the current work: (1) Can we identify the primary quenching routes in Yb:YLF NC samples and improve the PLQY by systematically removing the possible quenching routes? (2) Does concentration quenching in Yb:YLF disappear when we remove the quenching routes?

**Figure 1:**
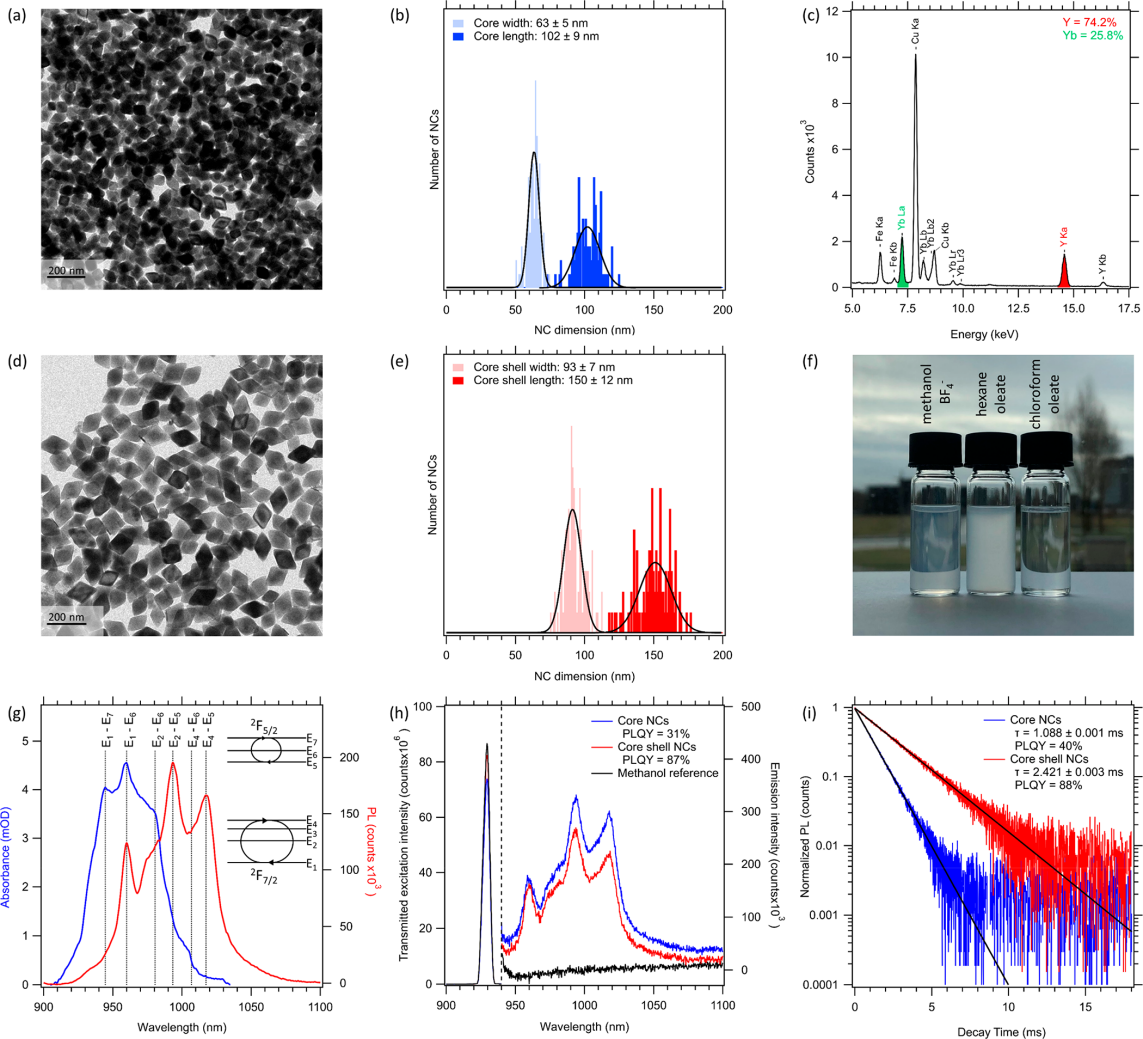
(a, d) TEM images and (b, e) size distributions of Yb(25%):YLF core and Yb(25%):YLF/YLF core/shell NCs, respectively. (c) EDX elemental analysis of the core NCs, showing the EDX peaks used for the determination of the Y:Yb ratio in red (Y) and green (Yb). (f) Yb(25%):YLF core/shell samples dispersed in different solvents showcasing the better dispersibility of NCs with BF_4_^–^ ligands, as well as the reduced scattering with index-matched chloroform. (g) Absorbance and emission spectra (λ_ex_ = 930 nm) of a Yb(50%):YLF core/shell NC sample dispersed in chloroform. The most prominent transitions are indicated in the spectra and correspond to the energy levels indicated in the top right. (h) PLQY analyses of Yb(30%):YLF core and Yb(30%):YLF/YLF core/shell NCs dispersed in methanol using an integrating sphere method and (i) a TRPL-based method, both excited at 930 nm.

Here, we systematically study the processes that reduce the PLQY in Yb:YLF NCs with a well-defined bipyramidal crystal shape and with a core size of ∼100 × 60 nm (Figure 1a) in an effort to understand and mitigate these processes. To reliably determine the PLQY of these NCs, we use *and verify* the optical model reported by Rabouw *et al*.^24,26^ This model allows one to extract the PLQY from the PL lifetime, correcting for the difference in refractive index between the particle and solvent. To remove the uncertainty from the latter correction, we used refractive index-matched chloroform as solvent. In addition, we verified the PLQY values obtained with two independent measurements based on the absolute emission of photons.

We first show that water plays a significant role in quenching the PL, likely through OH^–^ inclusion in the lattice during synthesis, but that this can be avoided by actively removing water through the addition of trifluoroacetic anhydride (TFAA) to the precursors before the synthesis, which has not yet been reported for the synthesis method we use. Next, we show that at low doping concentrations, the PLQY of these Yb:YLF NCs can be >90% but that at higher Yb^3+^ concentrations, significant concentration quenching occurs. This concentration quenching is completely eliminated by growing a 15 nm Yb^3+^ free YLF shell, allowing one to reach PLQY values of near unity even for cores consisting of pure YbLiF_4_. The PLQY in core-only samples is not dependent on the surface ligands and only weakly dependent on the solvent. Only when water or methanol is used, a slightly lower PLQY is observed for high Yb^3+^ concentrations. These results clearly show that all the processes that limit the PLQY in Yb:YLF NCs no longer happen inside the crystal but only take place at the surface; hence, all internal quenching is removed. Quenching is not related to the native oleate ligands or the BF_4_^–^ surfactants (after ligand removal). Also, a wide range of tested solvent molecules were not found to be responsible for PL quenching. Rather, we suggest that impurities are present on the surface that cause the PL quenching in core-only Yb:YLF NCs. We propose that OH^–^ ions are present as surface complexes, in line with the observation that the PLQY increases if the NCs are exposed to D_2_O in an effort to replace surface OH^–^ by OD^–^. Alternatively, it is possible that phonons at the YLF surface are involved in PL quenching. To understand the mechanism of Yb-Yb energy transfer, we modeled the PLQY of our NCs using Monte Carlo simulations. Using Förster resonance energy transfer (FRET), the PLQY trend can only be modeled if FRET is restricted to nearest-neighbor Yb ions. This short-range energy transfer suggests that Dexter-type energy transfer may be the dominant mechanism. Simulations employing a Dexter-type mechanism give a better agreement with the experimental data, although more complex quenching mechanisms cannot be excluded. We do conclude that energy transfer to the surface does not occur via FRET to localized Yb^3+^ ions. Our results shed light on the relevant quenching processes in Yb:YLF nanophosphors and offer a method to produce Yb:YLF NCs with very high Yb^3+^ content and a near-unity PLQY. These materials are promising for optical refrigeration and lasing applications.

## Results and Discussion

### Synthesis of Core and Core/Shell NCs Using Chemically Dried Precursors

Yb:YLF core and core/shell NCs were prepared *via* a modification of the synthesis reported by Yi *et al.*(27)Figure 1a shows that this synthesis results in well-defined tetragonal bipyramidal NC cores with a narrow size distribution (Figure 1b). Energy dispersive X-ray spectroscopy (EDX) measurements, shown in Figure 1c and Figures S1 and S2, indicate that the incorporation of Y and Yb follows the feed fraction of their respective precursors. XRD on core NCs (Figure S3) indicates that all NCs have the same crystal phase (scheelite structure) with a small lattice contraction for samples with higher fractions of Yb ions.

When shelling the NCs with a pure YLF shell, the expanded lattice of the shell does not result in any observable core/shell mismatch, as integrated differential phase contrast (iDPC) and scanning transmission electron microscopy (STEM) micrographs (Figure S4) display a continuous atomic lattice throughout the entire NC. TEM images of core/shell NCs indicate that the size distribution is furthermore not significantly affected by the shelling (Figure 1d,e).

A ligand exchange, following the general approach reported by Dong *et al*.,^28^ is performed to remove the oleate ligands and replace them with charge-balancing BF_4_^–^ anions. This allows the NCs to be dispersed and analyzed in polar solvents (Figure 1f and Figure S6). The samples absorb and emit in the near-infrared region (Figure 1g) *via* the parity-forbidden ^2^F_7/2_ → ^2^F_5/2_ transition of the Yb^3+^ ions. The different peaks result from crystal field splitting and are usually labeled E_1_–E_4_ (F_7/2_ ground-state levels) and E_5_–E_7_ (F_5/2_ excited-state levels) in the literature, as indicated in Figure 1g.^29^ As several of the 12 possible transitions overlap, we only indicate the most prominent transitions here.

Figure 1h shows a PLQY measurement using a calibrated integrating sphere (see Experimental Methods section for details) for core and core/shell NCs with 30% Yb^3+^ doping dispersed in methanol. The PLQY in this case is extracted from dividing the number of emitted photons (integrated PL spectrum minus background reference) by the number of absorbed photons (integrated reference excitation spectrum minus sample excitation spectrum around 930 nm). The extracted PLQY values are 31 and 87% for the core and core/shell NCs, respectively. By far the biggest uncertainty in this measurement lies in the small absorption fraction. This means that PLQY can only be determined for relatively high Yb^3+^ doping concentrations, where concentration quenching may significantly reduce the PLQY. Additionally, in the case shown in Figure 1h, the emission below 940 nm is not considered, resulting in an underestimation of the actual PLQY. Even for these relatively well absorbing samples containing 30% Yb^3+^, we assume that the error in PLQY is significantly larger than a general 3–5% error for these measurements.^30−33^

### Determination of the PLQY Using Time-Resolved Photoluminescence Spectroscopy

A faster and more reliable method to determine the PLQY was reported by Rabouw *et al.*(24,26) This method is based on fitting the time-resolved photoluminescence (TRPL) decay of the Yb^3+^ emission to extract the average PL lifetime (τ_PL_). The PLQY φ is obtained as φ = Γ_PL_/Γ_rad_ by comparing the measured PL rate constant (Γ_PL_ = 1/τ_PL_) to the radiative rate constant Γ_rad_, which itself is obtained from the bulk radiative rate constant Γ_rad_^bulk^ corrected for the different refractive indices of bulk YLF (*n*_YLF_) and NC dispersant (*n*):
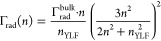
1

The approach is shown in Figure 1i, which reports TRPL measurements on the same Yb(30%):YLF core and Yb(30%):YLF/YLF core/shell NCs in methanol that were used for the integrating sphere measurement discussed above (Figure 1h). A single exponential fit is shown as the solid black lines and corresponds to a PL lifetime of 1.088 and 2.421 ms for the core and core/shell NCs, respectively. Following eq 1, this corresponds to PLQY values of 40 and 88%.

For the TRPL-based measurements, it is important that no significant reabsorption, or photon recycling, of light emitted by the NCs occurs, as this will increase the measured PL lifetime, which will overestimate the PLQY for the TRPL model.^23,34^ On the contrary, reabsorption leads to an underestimation of the PLQY for methods using an integrating sphere for any NC samples that do not have unity PLQY. To avoid errors in the estimation of the PLQY due to reabsorption, we have used dispersions with low concentrations of NCs, with <2% reabsorption at the emission wavelengths (Figure S7). Any applied corrections for reabsorption in the TRPL measurements show that the PLQY reduces by <1% for all samples (Figure S8). Furthermore, we have not observed any dependence of the PLQY on the used excitation or emission wavelengths (Figure S9) or on the NC concentration or used excitation fluence (Figure S10).

The largest uncertainty in estimating the PLQY from TRPL measurements comes from the correction for the refractive index difference between NC and solvent, *i.e.*, the last term in eq 1. This term is derived for *spherical* particles that are much smaller than the wavelength of the emitted light. Although in principle a further correction for the size of the particles can be applied (Supporting Information SI-9),^26^ the correct analytical shape factor for bipyramidal NCs is unknown. To circumvent possible errors that result from this, we have chosen to use refractive-index matched chloroform (*n*_CHCl3_ = 1.4359,^35^*n*_YLF_ = 1.4485^36^) for the TRPL-based PLQY measurements. Figure 1f shows a photograph of Yb(25%):YLF core/shell NCs dispersed hexane, chloroform, and methanol (after a ligand exchange to BF_4_^–^ counterions). The sample in chloroform is fully transparent, demonstrating successful index matching. For chloroform then, the last term in eq 1 approximates 1 even for other sizes and shapes.

The last remaining uncertainty in the application of eq 1 then lies in the correct value of the bulk radiative rate constant Γ_rad_^bulk^. In the literature, for the bulk lifetime, a variety of values between 2.0 and 2.2 ms can be found.^1,15,37,38^ Furthermore, previous reports have shown that the lifetime is temperature and Yb-concentration dependent.^1,4,37,38^ For the purpose of this work, we have chosen to use the bulk lifetime of 5% Yb:YLF, 2.20 ms, accurately reported by Püschel *et al.*(1) as fixed radiative lifetime, and used this throughout the rest of this work.

To further confirm the validity of the TRPL approach for determining PLQY values, we have compared the extracted PLQY values for a range of samples with the PLQYs obtained from two independent measurements using integrating spheres. The first of these, already mentioned above, employed a calibrated photoluminescence spectrometer designed to measure PLQY values. The second was performed using an absorption photospectrometer and requires solely the assumption that the transmission of the sphere and sensitivity of the detector are the same for the photons absorbed and emitted, which is reasonable as the difference in wavelength is small (see Figure 1g). The details of this measurement are explained in the Supporting Information (SI-10). All obtained PLQY values are summarized in Table 1, which shows that within 10%, all methods agree and yield the same PLQY value.

**Table 1:** PLQY Values Obtained from Integrating Sphere Measurements Compared to PLQY Values Obtained from TRPL Measurements on the Same Sample

sample	method	obtained PLQY (%)	method	obtained PLQY (%)	figure
Yb(30%)YLF methanol	integrating sphere: emission	31	TPRL	40	1h/1i
Yb(30%)YLF/YLF methanol	integrating sphere: emission	87	TRPL	88	1h/1i
Yb(50%)YLF chloroform	integrating sphere: absorbance	16	TRPL	13	S12a
Yb(50%)YLF/YLF chloroform	integrating sphere: absorbance	91	TRPL	96	S12b

The above discussion and comparison with other PLQY measurement methods show that the TRPL model is a useful, faster, and more easily applicable method than methods that rely on an accurately measured absorbance. For this reason, the TRPL method is used throughout the remainder of this work to study various PL quenching pathways in these Yb:YLF NCs.

### Overview of Potential Quenching Pathways in Yb:YLF

**Figure 2:**
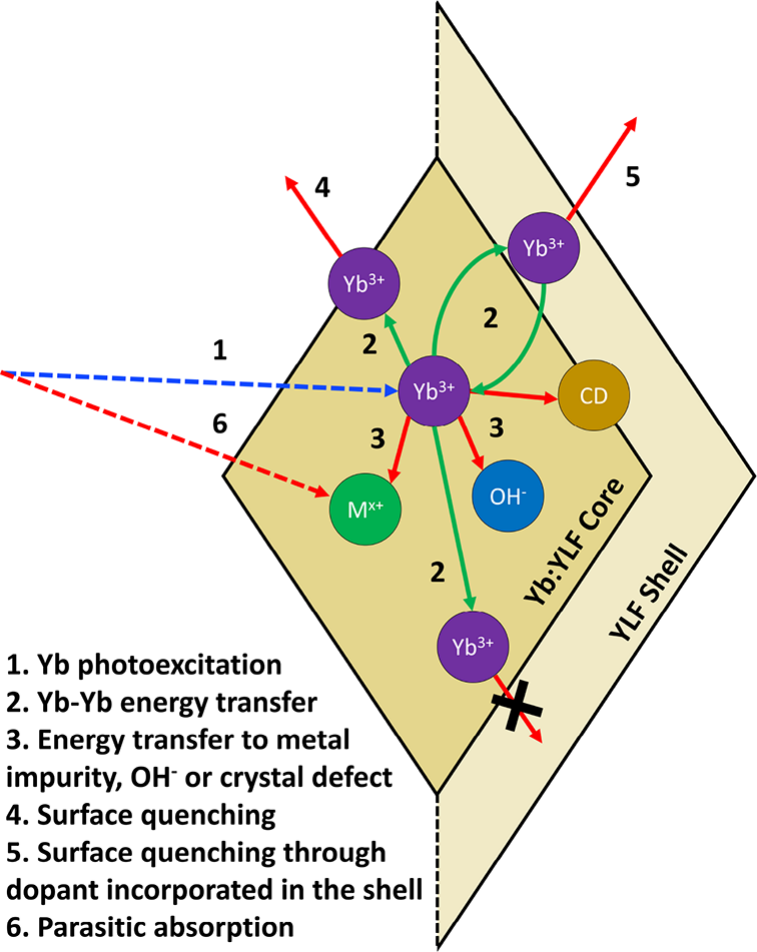
Photophysical pathways involved in photoluminescence and photoluminescence quenching. All red arrows indicate processes that result in a reduction of the PLQY, whereas the green arrows indicate energy transfer between Yb^3+^ sites.

An overview of possible quenching pathways is shown in Figure 2. From the literature, it is known that crystal defects and impurity ions (*e.g.*, d-block metals and lanthanides other than Yb^3+^) can reduce the PLQY via nonradiative recombination (process 3 in Figure 2) or parasitic absorption (process 6).^18^ We have attempted to reduce the PL-quenching influence of these factors as much as possible by using the highest possible temperature for the synthesis (reported to reduce the formation of crystal defects^39,40^) and the highest possible precursor purities.

### Effect of Water during the Synthesis on the PLQY of Core NCs

**Figure 3:**
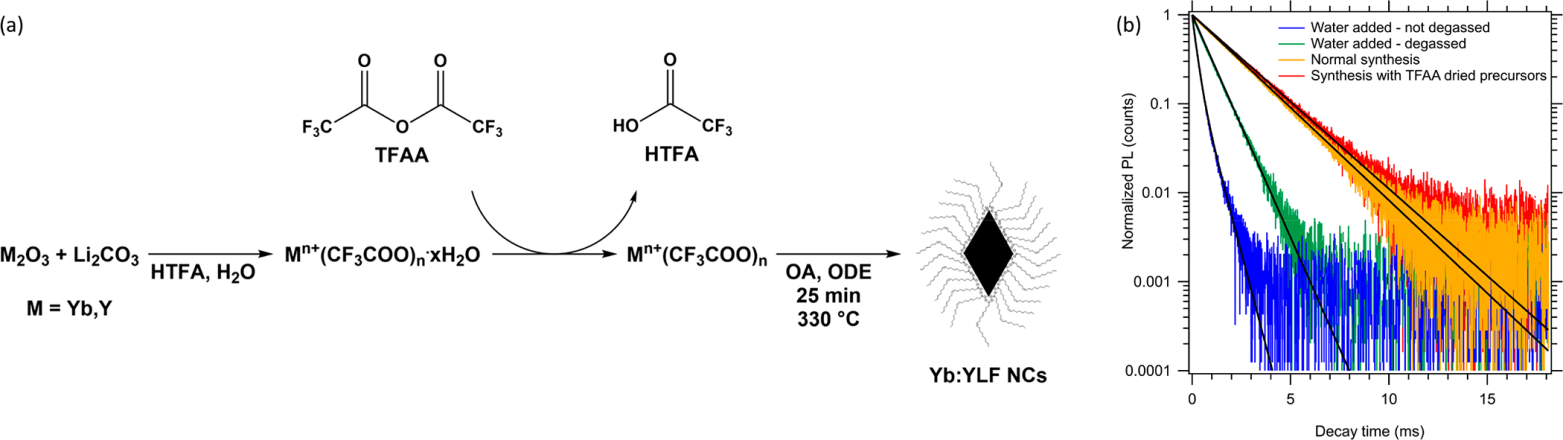
(a) A schematic representation of the synthesis of the TFA-based precursors from their oxides or carbonates with HTFA, the subsequent drying step utilizing TFAA, and the NC synthesis itself from the dried precursors. (b) TRPL spectra of Yb(10%):YLF core NCs dispersed in hexane (λ_ex_ = 930 nm). The blue and green curves correspond to samples where 250 μL of water was added before (green) or after (blue) the degassing step prior to the synthesis (see Experimental Methods and Supporting Information SI-11). The yellow and red curves correspond to samples synthesized with precursors that were (red) or were not (yellow) dried using TFAA.

It is reported that a main PL quenching pathway for Yb^3+^ emission is energy transfer to incorporated OH^–^ originating from water that is present during the synthesis.^24,25^ As OH^–^ ions substitute F^–^ ions in the YLF host, removing incorporated OH^–^ postsynthesis is difficult. Therefore, the synthesis should be performed completely water-free. As the trifluoroacetate (TFA) precursors for the NC synthesis are made using water, it is important to remove any water that is still present. To minimize the presence of internal OH^–^ impurities in the crystal lattice, we adopted a recent method by Homann *et al*.^41^ for the drying of metal acetate precursor salts and introduced it to our metal trifluoroacetate precursors (Figure 3a), which has not been reported before. To do this, we first synthesize the TFA salts from the respective metal oxide or metal carbonate and aqueous trifluoroacetic acid (HTFA), remove all free water and acid under vacuum, and subsequently add trifluoroacetic anhydride (TFAA) that chemically reacts away all traces of (crystal) water (see Figure 3a), irreversibly forming HTFA. Therefore, a significant improvement is made for this synthesis method, as the newly introduced drying method removes any traces of water that are left without introducing any chemical species that are not already present for the TFA salt synthesis. Subsequent removal of unreacted TFAA and any free HTFA is swiftly done by applying a vacuum, resulting in very dry and pure TFA salts. This is an overall improvement for Yb:YLF NC syntheses using TFA salts, for which until now no drying method existed. This drying method can furthermore be expanded to any syntheses relying on very dry TFA-based precursors.

The importance water has on the PLQY is shown in Figure 3b, where we show TRPL traces of NCs synthesized with water added and/or removed at different stages. The green and blue traces are obtained for samples where 250 μL of water (∼14 mmol) was added before (green) and after (blue) the degassing step that precedes the synthesis. In both cases, the PL lifetime is much shorter, and the extracted PLQY is much lower (31 and 8%, respectively), than without the addition of water. As shown in Figure S13, many small particles are present next to the YLF NCs, explaining the biexponential PL decay shown in Figure 3b. The yellow curve corresponds to a normal synthesis without the use of TFAA. The PL lifetime in this case is 2.08 ms, and the extracted PLQY is 79%. A further increase of the lifetime and PLQY (to 84%, τ = 2.22 ms) is observed for the samples synthesized with precursors that were additionally dried with TFAA. These results show the importance of removing water from the synthesis. To obtain the highest PLQYs, the TFA precursors need to be dried with TFAA.

### Disappearance of Concentration Quenching by the Growth of a Shell

The factors discussed so far (metal impurities, crystal defects, and OH^–^ incorporation) cannot be improved by postsynthesis procedures and therefore set an upper limit for the PLQY of Yb:YLF NCs. As we show later, these parameters have no significant influence on our NC samples because near-unity PLQY values can be obtained. Additionally, an important factor that reduces the PLQY of nanometer-sized phosphors is quenching at surface sites (Figure 2, process 4).^42,43^ This surface quenching can be mitigated by growing lattice matched shells of Yb-free YLF. This will prevent energy transfer to the surface and hence reduce surface-related PL quenching. It may inhibit surface quenching altogether if the shell is thick enough and Yb free. The latter is not trivial as Yb migration can potentially take place at the elevated temperatures usually employed to grow defect-free YLF shells, thereby introducing pathways for energy transfer to the NC surface (Figure 2, process 5).^44^

**Figure 4:**
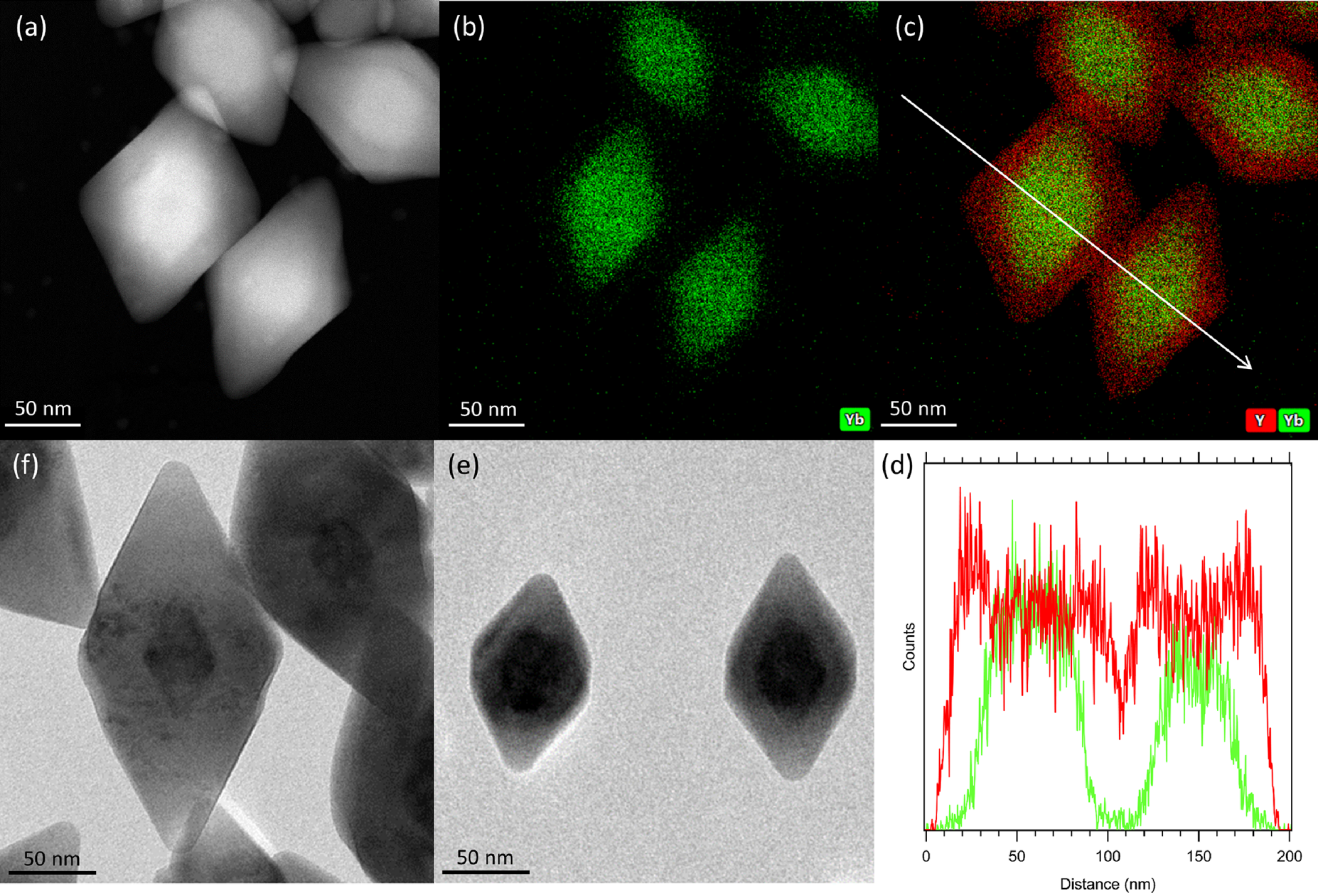
(a) HAADF-STEM image of Yb(25%):YLF/YLF core/shell NCs and (b) EDX analysis of Yb and (c) Y (red) and Yb (green) used to identify the Yb fraction in the YLF shell. The white arrow shows the direction of the line scan measurement shown in panel d. (d) EDX line scan of the NCs shown in panels a–c showing the intensity of Y (red) and Yb (green) through the NC shell and core. (e) TEM image of LiYbF_4_/YLF core/shell NCs and (f) LiYbF_4_/YLF/YLF core/shell/shell NCs showcasing a large contrast between the electron dense core and the less electron dense shell, as well as the possibility to grow additional layers of YLF in case Yb migration to the surface does affect the PLQY of the NC sample.

We synthesized Yb:YLF/YLF core/shell NCs by adding Y(TFA)_3_ and LiTFA to previously synthesized and purified Yb:YLF core NCs at the core synthesis temperature in a clean and hence Yb-free solvent mixture (see Experimental Methods section). Figure 4a shows a high-angle annular dark field-STEM (HAADF-STEM) image of the resulting Yb(25%):YLF/YLF core/shell NCs. The shell thickness of 15 nm can be estimated because of the difference in the atomic number of Y^3+^ (Z = 39) and Yb^3+^ (Z = 70). EDX was used to visualize the location of Y and Yb ions (Figure 4b,c and, including F^–^, Supporting Information SI-12). Only a very slight amount of Yb signal is recorded in the shell location, as well as in the exterior, potentially as a result of beam damage inflicted during the EDX measurements. An EDX line scan was performed (direction indicated in Figure 4c), confirming that the Yb signal quickly drops from the core to the shell (Figure 4d). This shows that no significant Yb^3+^ ion migration occurred during shell growth. This is likely due to our shelling method being much faster (45 min total) compared to the literature reporting ion migration (15 min per precursor addition plus a 2–12 h annealing step).^44^

**Figure 5:**
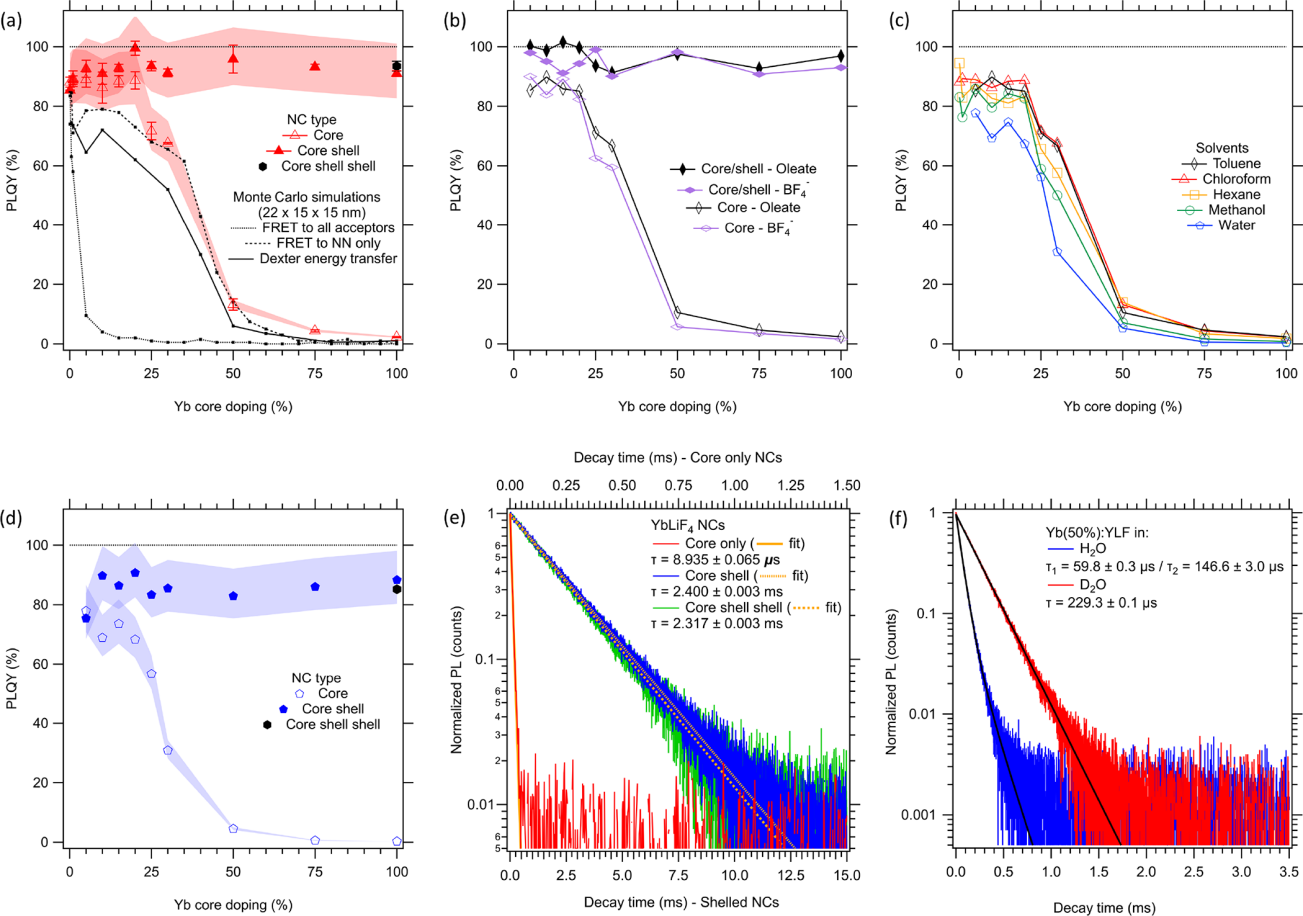
(a) PLQY values derived from PL lifetimes of core and core/shell NCs with different core Yb doping dispersed in index-matched chloroform. The error bars indicate the error of the PLQY value obtained by repeating the measurements. The red shaded area indicates the spread of PLQY when a 10% higher (2.42 ms) or lower (1.98 ms) bulk radiative lifetime is chosen than the 2.20 ms used in the rest of the analyses. The dotted, dashed, and solid black lines correspond to simulated PLQYs using Förster resonance ET or Dexter-type ET. (b) PLQY values calculated from PL lifetimes of core and core/shell NCs dispersed in toluene with either oleate or BF_4_^–^ ligands and (c) core NCs dispersed in different solvents. (d) PLQY values derived from PL lifetimes of core and core/shell NCs with different core Yb doping dispersed in water. Similar to panel a, the influence of a 10% change of bulk PL lifetime is indicated by the blue shaded area. (e) TRPL spectra of 100% LiYbF_4_ core (red), core–shell (blue), and core–shell–shell NCs (green) dispersed in water. (f) TRPL spectra of Yb(50%):YLF NC cores dispersed in H_2_O (blue) and D_2_O (red) showcasing a longer PL lifetime and single exponential PL decay when D_2_O is used.

The thickness of the YLF shell can in principle be increased at will by repeating the shelling procedure. Figure 4e,f demonstrates this for pure LiYbF_4_ core NCs with a single shell of 15 nm (Figure 4e) and an increased shell thickness of 30 nm. In the remainder of this work, we employed only core–shell NCs with a 15 nm shell, as we will later discuss that this is sufficient to remove the effect of surface quenching (Figure 5a,d,e, *vide infra*), in accordance to Rabouw *et al*.^24^

To investigate the effect of surface-related quenching, we studied the Yb^3+^ concentration-dependent PLQY. A set of core-only NC samples with different degrees of Yb doping was synthesized (using the extra dried precursor method) and dispersed in index-matched chloroform. The PLQY was determined from the PL lifetime using eq 1. The results are shown as the open symbols in Figure 5a. At low doping densities, the PLQY is high (80–90%). However, from Yb(25%):YLF onward, the PLQY rapidly reduces with increasing doping density and drops to nearly 0 for >75% Yb^3+^. The observed concentration quenching can be attributed to efficient energy transfer (ET) between Yb^3+^ ions, allowing the excitation to reach quenching sites located on the surface within the radiative lifetime of the Yb ion, as often reported in the literature.^45−47^

The solid symbols in Figure 5a represent samples where a 15 nm Yb-free YLF shell was grown around the same Yb:YLF core-only NCs. In this case, the PLQY is even higher (>90% at low Yb concentration, with 99.5 ± 2.4% at 20% Yb as the highest obtained value). Strikingly, for these core/shell NCs, there is no sign of concentration quenching even up to “100% Yb-doped” LiYbF_4_/LiYF_4_ NCs. In that specific case, the PLQY of the core-only sample is only 2% (τ = 50 μs), whereas the extracted PLQY of the core/shell sample is 91% (τ = 2.04 ms). The addition of a second shell increased this PLQY even further to 94% (τ = 2.10 ms). To show the uncertainty of the reported values, the average PLQY of the samples is plotted (as markers) together with the measurement error from performing multiple measurements (error bars), indicating the generally high reproducibility of the measurements. The influence of a deviation from the used bulk radiative lifetime (2.20 ms) is shown by the red shaded area. Here, a PLQY range is shown to indicate the accuracy of the measured PLQY when using a 10% increase or decrease of the bulk lifetime.

The clear concentration quenching of the core-only samples and the absence thereof in the core/shell NCs clearly demonstrate that the main PL quenching occurs on the surface. At low Yb concentrations, this results only in a marginal decrease of the PLQY in core-only NCs because photoexcitation predominantly takes place in the interior of the NCs. At higher Yb concentrations (>20%), efficient ET causes the photoexcitation to migrate through the NCs to the surface, where PL quenching takes place. An Yb-free YLF shell prevents migration of the excitation to the surface.

### Modeling the PLQY via Yb-Yb Energy Transfer to the Surface

To confirm the above hypothesis and investigate the ET process, we simulated PL quenching by a full Monte Carlo simulation. Here, bipyramidal Yb:YLF NCs of varying sizes are modeled atomistically, with a stochastic distribution of Yb^3+^ ions in the lattice. The details of this approach are reported in the Supporting Information (SI-14). Initially, we considered that migration of the excitation occurs *via* FRET using , where (48) and *R*_Yb_ is the distance to the other Yb atoms. The Förster radius *R*_0_ was estimated to be 1.5 nm on the basis of a similar value reported for FRET in Er^3+^/Yb^3+^:NaYF_4_.^49^ We assume that efficient quenching at the surface takes place. This means that any excitation that migrates to the first two monolayers of the NC surface (see Supporting Information for details) is removed from the simulation, reducing the PLQY. No other sources of PL quenching are included.

The black lines in Figure 5a show the PLQY as predicted by the MC simulations for a 22 nm large NC. The dotted black line, which assumes that FRET can occur to all other Yb^3+^ ions, predicts that concentration quenching already sets in at low concentrations. Regardless of the parameters used in the calculations, we do not find a plateau with constant PLQY values in combination with near-zero PLQY values at the highest Yb^3+^ concentrations, as is observed in the experiment. However, if we only allow FRET to nearest-neighbor Yb ions, the obtained trend for PLQY vs Yb concentration, as given by the dashed black line, agrees well with the experimental data points. The simulated PLQY value in the low concentration plateau increases with increasing NC size (see Figure S16a), from ∼50% at a long NC axis length of 10 nm to ∼90% for 68 nm, simply reflecting the surface to volume ratio of the NCs. The reason that the simulated PLQY values in Figure 5a are lower than the experimental values is due to the smaller size of the simulated NCs (22 × 15 × 15 nm) vs the experimental NCs (100 × 60 × 60 nm).

The observation that FRET to nearest-neighbor Yb^3+^ ions reproduces the experimental data shows that the range of the ET process is shorter than expected for FRET, suggesting that ET may be mediated via the exchange interaction rather than via electric dipole coupling, and hence may correspond to Dexter-type energy transfer (DET). There are however only few literature references that discuss DET in similar lanthanide-doped systems.^50−52^

We simulated DET in a manner identical to the FRET simulations discussed above but using a transfer rate constant *k*_Dexter_ = *A* × exp ( – β × *r*) (see Supporting Information SI-14 for details). The distance dependence is mostly governed by the tunnel decay parameter β, whereas the prefactor *A* sets the overall rate constant. The solid black line in Figure 5a is the result of such a simulation. As can be seen, it gives similar results to FRET using nearest neighbors only and captures the trends seen in the experimental data. As above, the PLQY value in the plateau is artificially low because of the small simulated crystal size. We remark that the solid black line was obtained with *A* = 2.38 × 10^8^ μs^–1^ and β = 0.5 nm^–1^. This tunnel decay parameter is higher than the value of 0.1 nm^–1^ that is more commonly used.^52^ Lower values of β fail to reproduce the PLQY plateau at low Yb^3+^ concentrations. This shows that the ET mechanism must have a very steep distance dependence, but raises questions about the details of the DET simulations. The model used here is clearly an oversimplification. Finite surface quenching rates or a distribution of quenching sites on the surface could improve the model and perhaps allow the data to be described with more reasonable DET or FRET parameters.

Overall, the simulations validate that the Yb^3+^ concentration dependence of the PLQY is explained by energy transfer to the surface and suggest that Dexter-type energy transfer may be the dominant ET mechanism responsible. To understand the complete ET mechanism and concomitant quenching, modeling is required to distinguish between all the possible scenarios based on global fits to all the PL-decay curves rather than the PLQY trend that we have modeled. The main important observation here is that FRET alone does not describe the PLQY trends observed.

### Effect of Surface Ligands, Solvents, and Unwanted Surface States on the PLQY

In an attempt to identify the nature of surface PL quenching, we measured the PLQY with different surfactants and in different solvents. As mentioned before, from all samples shown in Figure 5a, the native oleate ligands were reacted away and replaced by BF_4_^–^ ligands following the procedure reported by Dong *et al*.^28^ This allowed stable dispersion of the NCs in polar solvents (Figure 1f). Both the oleate- and BF_4_^–^-containing NCs were dispersed in toluene (as the BF_4_^–^-containing NCs do not disperse well in chloroform); the PLQYs were determined and are shown in Figure 5b. It is clear that the absolute PLQY values as well as the dependence on the Yb^3+^-doping fraction are nearly identical for both surface molecules. Therefore, we conclude that the oleate surface ligands and BF_4_^–^ counterions do not have a large influence on the overall PLQY of the NCs.

To identify the influence of ET to the surrounding solvent molecules on the PLQY, the PLQYs of NC samples dispersed in toluene, chloroform, and hexane (with oleate ligands) and in toluene, methanol, and water (with BF_4_^–^-ligands) were compared. As shown in Figure 5c and Figure S17, the same concentration quenching trend is again clearly visible, and similar PLQY values of >80% are observed at low Yb^3+^ concentrations. This observation suggests that energy transfer to solvent vibrations is not the limiting factor for the PLQY. Indeed, energy transfer to overtones of C–H vibrations in, *e.g.*, hexane, toluene, and chloroform is reported to be roughly 15 times slower, and energy transfer to overtones of C–C and C–Cl vibrations is orders of magnitude slower than energy transfer to −OH vibrations in water or methanol.^53,54^ This suggests that there is an additional source of PL quenching at the surface of the NCs. The samples in nonpolar solvents contain oleate surface ligands, which could potentially induce quenching by energy transfer to vibrations of the biding carboxylate group. In addition, we consider that there possibly could be OH^–^ groups binding to the surface that could act as quenchers. In the case of methanol and water, these oleate ligands have been removed, but here, the solvent −OH groups could act as quenchers for the core-only Yb:YLF NCs.^55−57^

To highlight the PLQY in water, which is the most important solvent for applications in, *e.g.*, biological systems, we show in Figure 5d how the PLQY of core-only and core–shell Yb:YLF NCs depends on Yb^3+^ doping concentration when dispersed in water (after ligand removal with [Et_3_O][BF_4_]). For the core-only samples, we observe concentration quenching for concentrations of >20%. The core–shell samples do not show concentration quenching even up to 100% Yb^3+^, similar to the observation made for samples in chloroform (Figure 5a). Indeed, this is expected for the relatively thick shell of 15 nm that was used in this work, which should slow down energy transfer to solvent or surface states by several orders of magnitude.^24^ To further exclude the effect of energy transfer to the solvent or surface, we also increased the shell thickness to 30 nm (black solid marker in Figure 5d) and found that the observed PL lifetime is the same, within the measurement uncertainty, as for the 15 nm shells (green and blue lines in Figure 5e).

Although these experiments exclude quenching via energy transfer to solvent vibrations in the core–shell samples, the PLQY in water does appear to be lower than that in chloroform. The average PLQY of the core–shell NCs for all Yb concentrations in water is 86 ± 4%, whereas in chloroform, we find 93 ± 3%. We consider however that accurate PLQYs in water are more difficult to determine exactly as water has a much larger mismatch of refractive index with YLF than chloroform, making the correction to the radiative lifetime via eq 1 much larger and more prone to errors than in index-matched chloroform. We show in Figure S18 and the related discussion in the Supporting Information that the PLQY derived via eq 1 decreases systematically with decreasing refractive index of the solvent. We therefore trust the derived PLQY values in chloroform better and consider the values reported here for water to be a lower limit of the real PLQY.

As indicated before, quenching on the surface can also be due to unintentional surface molecules, which in that case are potentially equally present in all solvents, depending on their origin. The usual candidate in the literature is surface OH^–^ originating from water.^21,24,25,58^ In principle, this should be removed by the ligand exchange procedure as OH^–^ is expected to react with triethyloxonium cations, which otherwise reacts with surface oleate ligands (see Experimental Methods), to form diethyl ether and ethanol. To test the role of OH^–^ in the observed PLQY quenching at the surface, an Yb(50%):YLF core NC sample dispersed in water was washed thrice and resuspended each time in pure D_2_O. As protons readily exchange with deuterons, surface OH^–^ is expected to convert to OD^–^. The vibrational energy of the OD^–^ stretch is about √2 times smaller than the OH^–^ vibrational stretch energy, lowering the probability of PL quenching via energy transfer to OD^–^. As shown in Figure 5f, the PL lifetime of the sample in D_2_O is significantly longer and hence the PLQY is larger than in H_2_O. This is in accordance with previous observations on Yb:NaYF_4_^25,59^ and with the notion that surface quenching may involve adsorbed OH^–^. However, because the role of the solvent in the quenching process cannot be excluded here, we cannot definitively conclude that surface OH^–^ species are the dominant PL quencher. Alternatively, it is possible that phonons at the YLF surface are involved in PL quenching. It is well known that the phonon modes at the NC surface differ from bulk phonon modes, have a much broader spectrum, and extend to higher frequencies.^60^ The maximum bulk optical phonon energy in YLF is 560 cm^–1^ (69 meV),^61,62^ which implies that multiphonon relaxation to the ground state involves on average 18 phonons (λ_em_® = 992 nm, *E*_em_® = 10,082 cm^–1^ = 1.250 eV, Supporting Information SI-17) and is inefficient. Higher energy surface phonon modes could potentially speed up nonradiative multiphonon emission to the ground state.

## Discussion

Yb:YLF NCs synthesized with chemically dried precursors have shown high PLQYs (>80%) for all samples with less than 25% Yb doping. Above this concentration, a rapid PLQY decrease is observed, which we attribute to efficient ET to the NC surface, where the photoexcitation is rapidly lost, reducing the PLQY for higher Yb-doped cores. Shelling these cores with an Yb-free YLF shell has shown to yield high PLQYs for all Yb doping fractions. The PLQY of Yb(20%):YLF/YLF NCs is practically unity (99.5 ± 2.4%), and as it is measured in index-matched chloroform, the only remaining insecurity is the correct assessment of the bulk lifetime. Even for “100% doped” LiYbF_4_/LiYF_4_ NCs, the obtained PLQY is above 90%, providing improved opportunities for applications where a higher absorption cross section is preferred, for example, in biomedical imaging or in optically pumped lasing media. NCs with PLQYs >96.3% (Supporting Information SI-17) can furthermore exhibit optical refrigeration when excited at the right wavelength. On the basis of their PLQY values, some of the samples reported here should show optical refrigeration (as a NC ensemble); however, this is within the uncertainty of the PLQY measurements. We have tried to measure optical refrigeration in solutions and on films by analyzing the change of emission peak ratios as mentioned by Luntz-Martin *et al*.^63^ (influenced by the number of phonons present, Supporting Information SI-18 and SI-19), but the results do not yet convincingly show evidence of cooling, possibly as a result of too efficient heat transfer from the surroundings to the NCs. Efforts to measure optical refrigeration on single NCs using optical levitation, as reported before,^15,64−66^ are currently under way.

## Conclusions

We have reported an optimized protocol for the synthesis of large Yb:YLF NCs to reach near-unity quantum yields even for LiYbF_4_/LiYF_4_ core/shell NCs. We showed that any water present during the synthesis has a strongly negative effect of the PLQY but that we can remove all water from our precursors by extensive drying procedures using trifluoroacetic anhydride. Next, we showed that growing an undoped YLF-shell around the Yb:YLF core is essential to obtain high PLQYs even for full YbLiF_4_-based core shell samples. We conclude that all internal quenching is removed and hence the main cause of PL quenching lies on the surface of the NCs. We show that this is not related to the intentional surface molecules (oleate or BF_4_^–^) or the solvent but is most likely due to unintentional surface moieties (*e.g.*, OH^–^) or surface phonon modes. Numerical simulations confirm that the Yb^3+^ concentration dependence of the PLQY is explained by energy transfer to the surface and suggest that Dexter-type energy transfer may be the dominant ET mechanism or that more complex quenching mechanisms are predominant, and confirm that FRET is not the main route for energy transfer.

## Experimental Methods

### Materials

1-Octadecene (ODE, technical grade, 90%), triethyloxonium tetrafluoroborate ([Et_3_O][BF_4_], ≥97.0%), acetonitrile (ACN, 99.8%, anhydrous), chloroform (≥99%, anhydrous), and methanol (≥99.9%, anhydrous) were purchased from Sigma-Aldrich. Oleic acid (extra pure), trifluoroacetic acid (HTFA, ≥99.0%, for HPLC), trifluoroacetic anhydride (TFAA, ≥99.0%), lithium carbonate (Li_2_CO_3_, 99.999%, trace metal basis), yttrium oxide (Y_2_O_3_, 99.9999%, REO), and ytterbium oxide (Yb_2_O_3_, 99.998%, REO) were purchased from Fisher Scientific. Toluene (≥99.8%, anhydrous) and ethanol (≥99.8%, anhydrous) were purchased from VWR Chemicals. Hexane (>96.0%, anhydrous) was purchased from TCI. Deuterium oxide (D_2_O, >99.90% D) was purchased from Fluorochem Ltd.

All chemicals were used as received unless specified differently. All manipulations were performed under a N_2_ atmosphere using standard Schlenk line techniques or a nitrogen-filled glove box (<0.1 ppm H_2_O; <0.1 ppm O_2_) unless otherwise mentioned.

Milli-Q water was obtained from a Milli-Q Advantage A10 system (Merck Millipore, 18.2 MΩ·cm, 2 ppb TOC).

### Synthesis and Drying of Metal Trifluoroacetate Precursors

Metal trifluoroacetate salts, M^*x*+^(TFA)_*x*_ (M = Li^+^, Y^3+^, Yb^3+^), were synthesized by adding 5 mmol Li_2_CO_3_ (369 mg), Y_2_O_3_ (1129 mg), or Yb_2_O_3_ (1970 mg) and 5 mL of Milli-Q water to a 25 mL two-necked flask with a fused thermocouple insert containing a PTFE-coated stirring bar. The flask was connected to a Schlenk line equipped with a water-cooled condenser, and 5 mL of trifluoroacetic acid (HTFA; ∼65 mmol) was added dropwise under stirring. *Note: As the reaction between HTFA and Li_2_CO_3_ is strongly exothermic and results in the release of a large volume of CO_2_, the acid should be added carefully to avoid splashing.* After all of the acid had been added, the necks of the flask were closed with septa, and the reaction mixture was placed under N_2_ gas flow. The mixture was heated to 120 °C and left under reflux until a clear, colorless solution was obtained (generally <1 min for Li_2_CO_3_ and >1 h for Y_2_O_3_ and Yb_2_O_3_). At this point, the reaction mixture was cooled to below 50 °C, and a vacuum was applied to evaporate all water and acid. The resulting solids were transferred to a glove box and crushed to form a white powder. These powders may appear dry but are very likely to contain crystal water, especially in the case of lithium trifluoroacetate, which is extremely hygroscopic.

To remove the undesired crystal water, the precursor salts were dried further using trifluoroacetic anhydride (TFAA). Briefly, the powdered metal TFA salts were added to a 250 mL round-bottom flask, connected to a Schlenk line, and placed under nitrogen flow. TFAA (5 mL, ∼36 mmol) was added, resulting in a rise in the temperature of the reaction mixture. This mixture was left to stir for 1 h, after which the TFAA and HTFA were removed under a vacuum. The remaining solids were transferred back to a nitrogen-filled glovebox and crushed to a powder. *Note: TFAA reacts aggressively with water and can damage plastic and rubber tubing. To protect the Schlenk line tubing and the vacuum pump, the evaporated TFAA was collected in an additional cold trap. The cold trap contents were carefully quenched with isopropanol before disposal.*

### Synthesis of Yb:YLF Core Nanocrystals

Yb:YLF core nanocrystals were synthesized according to a protocol modified from the protocol reported by Yi *et al*.^27^

To a two-necked round-bottom flask (25 mL) with a fused thermocouple insert, 2 mmol of LiTFA (240 mg) and 2 mmol of a mixture of YTFA_3_ (428 mg/mmol) and YbTFA_3_ (515 mg/mmol) in the ratios required were added inside a nitrogen-filled glovebox. To this, a mixture of 6.5 mL of ODE and 6.5 mL of OA, both previously degassed, was added. The flask was then attached to a Schlenk line without exposure to the air, and the contents were degassed at 100 °C for 1 h. Thereafter, the flask was put under a flow of N_2_, and the temperature was increased stepwise to 330 °C with increments of 5 °C/30 s. Once the contents reached 330 °C, the reaction was left at this temperature for 25 min and subsequently cooled down to room temperature using compressed air. The resulting mixture was yellow and opaque at high temperatures but became transparent again at <100 °C. Using a syringe, 2 mL of anhydrous toluene was added to facilitate transfer from the flask to a nitrogen-filled vial. Anhydrous ethanol (∼10 mL) was added to the crude NC mixture until it turned slightly opaque. Addition of too much ethanol generally results in the presence of smaller NCs in the sample as a byproduct. The mixture was centrifuged at a relative centrifugal force of 1800*g* (3800 rpm) for 10 min, the supernatant was discarded, and the solid NC pellet was redispersed in 2 mL of anhydrous hexane. To this, 1 mL of anhydrous ethanol was added, and after centrifugation, the NC pellet was redispersed in 2 mL of anhydrous hexane. This step was repeated once more, and the final NC sample, dispersed in 2 mL of anhydrous hexane, was stored in a nitrogen-filled glovebox.

For the syntheses shown in Figure 3b, 250 μL (∼14 mmol) of Milli-Q water was added before (green trace) or after (blue trace) the degassing step at 100 °C for 1 h. For the yellow trace, TFA precursors were used as described but without the additional drying step with TFAA.

### Synthesis of Yb:YLF/YLF Core/Shell Nanocrystals

A shell precursor mixture was prepared by adding 2 mmol LiTFA (240 mg) and 2 mmol YTFA_3_ (856 mg) to 5 mL of ODE and 5 mL of OA, both previously degassed. While stirring, the mixture was heated up, and once the salts were completely dissolved, the shelling mixture was loaded into a 20 mL syringe.

On a Schlenk line, a two-necked flask with a glass thermocouple insert was degassed and filled with dry nitrogen. To this flask, 1 mL of the Yb:YLF core NCs (half a batch) and 13 mL of a previously degassed mixture of ODE and OA (1:1 ratio) were added, and hexane was removed by applying a vacuum at room temperature for 5 min. The mixture was placed under nitrogen flow and heated quickly (<2 min) to 330 °C. When the temperature reached 100 °C, the injection of the shelling precursors was started at a rate of 0.5 mL/min using a syringe pump. When the injection was completed (20 min), the solution was kept at 330 °C for another 25 min before it was cooled down quickly using a compressed air flow on the outside of the flask. The NCs were purified in an identical manner as the NC cores by washing the sample three times with ethanol and redispersing in hexane. Ultimately, the NCs were redispersed in 4 mL of hexane and stored inside a glovebox.

### Ligand Exchange

To disperse the NCs in polar solvents, the nonpolar oleate ligands were exchanged for tetrafluoroborate anions (BF_4_^–^) following the procedure adapted from Dong *et al*.^28^ In short, inside of a nitrogen-filled glovebox, 2 mL of a ∼100 mM solution of triethyloxonium tetrafluoroborate ([Et_3_O][BF_4_]) in acetonitrile (ACN) (20 mg/mL) was added to 1 mL of the stock solution of NCs in hexane. This solution was shaken and left standing for 5 min to react. Subsequently, the particles were directly centrifuged without the addition of an antisolvent. Then, the supernatant was discarded, and 3 mL of the 10 mM solution of [Et_3_O][BF_4_] in ACN was added to the solid pellet. Using a vortex, the NCs were dispersed in the solution, and 5 min later, the NCs were centrifuged and the supernatant was discarded. This was performed once more, for a total of three exchange steps, and subsequently, the NCs were dispersed in 4 mL of methanol.

### Optical Characterization

Absorption spectra were recorded on a PerkinElmer Lambda 1050 with an integrating sphere. All samples for the shown absorbance measurements were dispersed in chloroform.

Emission spectra were obtained using an Edinburgh Instruments FLS980 spectrometer equipped with a liquid-nitrogen-cooled NIR PMT-based detector from Hamamatsu. A Xenon arc lamp from XBO was used as the excitation source.

TRPL spectra were also obtained using an Edinburgh Instruments FLS980 spectrometer equipped with a liquid-nitrogen-cooled NIR PMT-based detector from Hamamatsu. The measurements were performed using time-correlated single photon counting, with 930 nm nanosecond laser pulses from a M8903-01 Hamamatsu laser unit as the excitation source.

PLQYs were determined using an Edinburgh Instruments FLS980 spectrometer with a calibrated integrating sphere. Measurements were performed using identical 1 × 1 cm quartz cuvettes. The emission was recorded in a 900–1100 nm window, where the samples were excited at 930 nm. The emission was recorded for 1 s per 0.25 nm, and the full measurement was repeated three times. The used excitation and emission slits were set at 5 and 2 nm, respectively.

Furthermore, PLQYs were determined through absorbance measurements using a PerkinElmer Lambda 1050 with an integrating sphere. The method is fully explained in Supporting Information SI-10. The spectra were recorded in a 900–1035 nm window. The absorbance was recorded for 0.5 s with a step size of 0.1 nm.

### Structural Characterization

TEM and ED images were acquired using a JEOL JEM1400 transmission electron microscope operating at 120 kV, equipped with an SSD-EDX detector for spot (>75 nm) analysis.

High-angle annular dark field scanning transmission electron microscopy images and energy dispersive X-ray spectral maps were acquired using an aberration-corrected cubed Thermo Fisher Scientific-Titan electron microscope operated at an acceleration voltage of 300 kV equipped with a Super-X detector.

XRD measurements were performed with a Bruckner D8 ADVANCE diffractometer (Cu Kα, λ = 0.15406 nm). The NC samples were dropcasted on zero diffraction (911) silicon substrates.

## Supporting Information Available

EDX analysis core NCs, XRD of core NCs, single NC iDPC and STEM, NCs in different solvents, influence of reabsorption, PLQY correction for reabsorption, measurement dependencies, PLQY from integrating sphere (absorbance), TEM and ED of samples synthesized with water, ensemble EDX measurements, EELS elemental analysis, numerical PLQY simulations, PLQY of NCs in different solvents, effect of refractive index on average PLQY, extended TRPL model, PLQY for optical refrigeration, temperature-dependent emission spectra, and temperature of NCs from their emission spectra (PDF)
