# Supplementary material for: Understanding and Preventing Photoluminescence Quenching to Achieve Unity Photoluminescence Quantum Yield in Yb:YLF Nanocrystals
Source: ACS Appl Mater Interfaces. 2023 Jan 6;15(2):3274–86. doi: 10.1021/acsami.2c17888 (PMC9869336; doi:10.1021/acsami.2c17888)
Supplement: Supplementary file 1 — am2c17888_si_001.pdf [file am2c17888_si_001.pdf]

Supporting information for

Understanding and Preventing Photoluminescence Quenching to Achieve Unity Photoluminescence Quantum Yield in Yb:YLF Nanocrystals

Jence T. Mulder[†], *Michael S. Meijer*[†], *J. Jasper van Blaaderen*[†], *Indy du Fossé*[†],
Kellie Jenkinson[‡], *Sara Bals*[‡], *Liberato Manna*[§], *Arjan J. Houtepen*[†]*

† Optoelectronic Materials Section, Faculty of Applied Sciences, Delft University of Technology, van der Maasweg 9, 2629HZ Delft, The Netherlands

‡ Electron Microscopy for Materials Science (EMAT), Department of Physics, University of Antwerp, Groenenborgerlaan 171, 2020 Antwerp, Belgium

§ Department of Nanochemistry, Istituto Italiano di Tecnologia (IIT), Via Morego 30, 16163 Genova, Italy

**Address correspondence to A.J.Houtepen@tudelft.nl*

Contents

SI-1 – EDX on Yb:YLF Core NCs S-1

SI-2 – XRD of $LiYF_4$, Yb(50%):$LiYF_4$ and $LiYbF_4$ Core NCs S-3

SI-3 – Crystallinity of Yb(25%):YLF/YLF Core/Shell NCs S-4

SI-4 – Index Matching of NCs with Solvent S-5

SI-5 – Influence of Reabsorption S-6

SI-6 – PLQY Correction for Reabsorption S-7

SI-7 – Excitation and Emission Wavelength Dependencies S-8

SI-8 – Concentration and Fluence Dependency S-9

SI-9 – Extended TRPL-Model S-10

SI-10 – PLQY from Integrating Sphere (Absorbance) S-11

SI-11 – Syntheses With Water – TEM and ED Analysis of Samples S-12

SI-12 – EDX of Yb(25%):YLF/YLF Core/Shell NCs – Lower Magnification S-13

SI-13 – EELS Analysis of Yb(25%):YLF/YLF Core/Shell NCs S-14

SI-14 – Förster Resonance- and Dexter-Type Energy Transfer Simulations S-15

SI-15 – PLQY of NCs in Different Solvents S-18

SI-16 – Effect of the Refractive Index on the Average PLQY S-19

SI-17 – PLQY for Optical Refrigeration S-20

SI-18 – Temperature Dependent Emission Spectra S-21

SI-19 – Temperature of NCs from Emission Spectra S-22

References S-24

SI-1 – EDX on Yb:YLF Core NCs

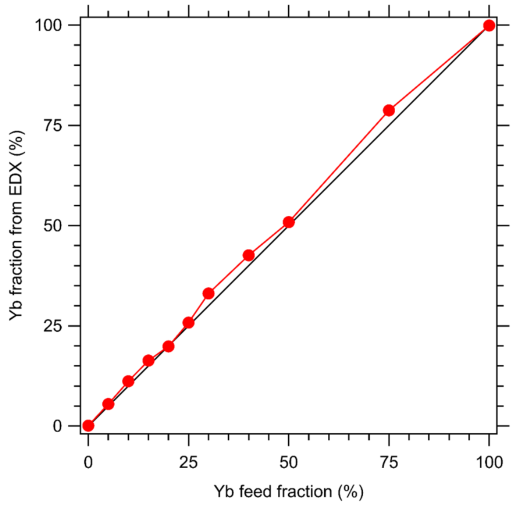

Yb feed fraction (%)	Yb fraction from EDX (%)	Yb feed fraction (%)	Yb fraction from EDX (%)
0	0.0925	25	25.8
5	5.48	30	33.1
10	11.2	50	50.8
15	16.3	75	78.7
20	19.9	100	100

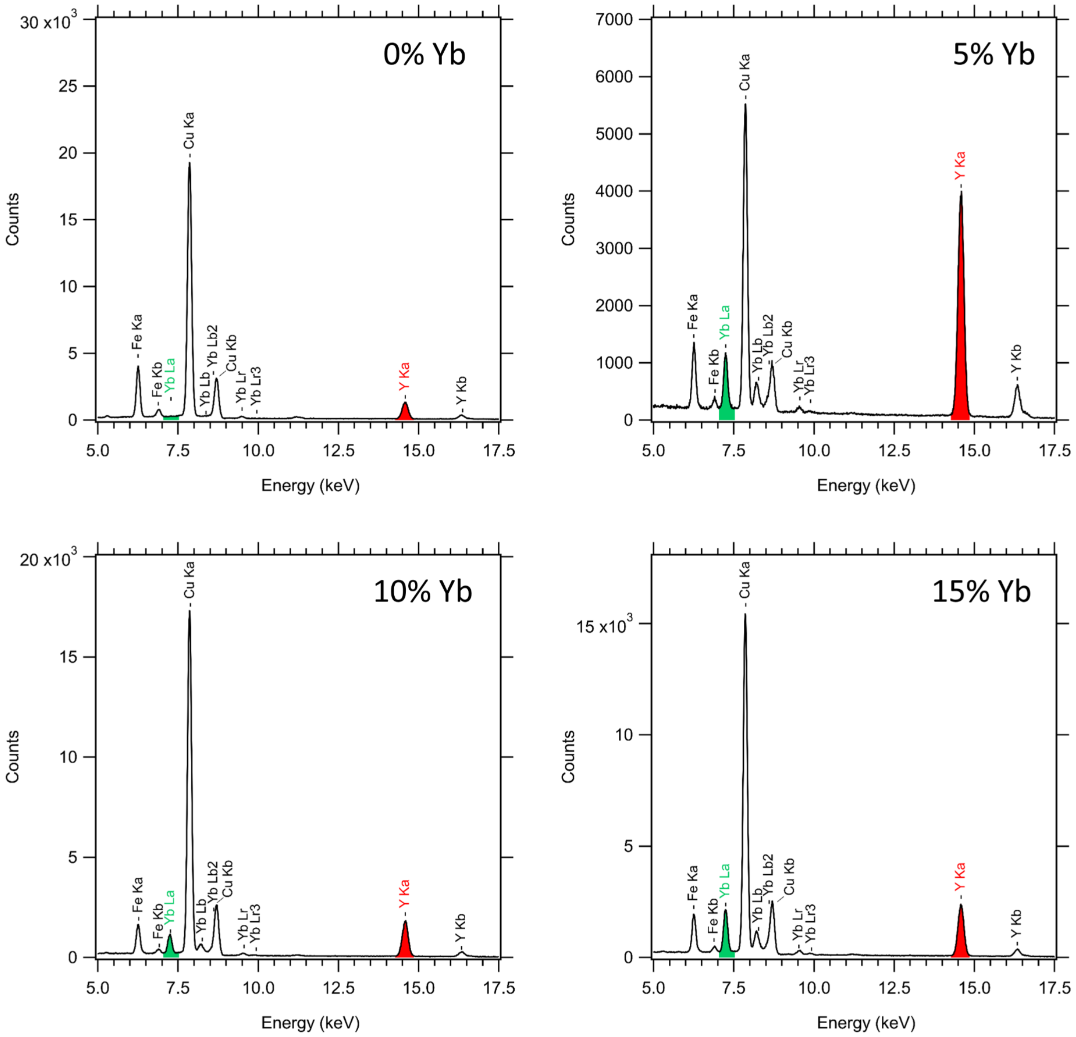

Figure S1. EDX measurements performed on the core NCs (doping concentration 0-15 %) indicate that the incorporation of Y (red) and Yb ions (green) is the same as the intended weighed out ratio. Therefore, the Yb core doping percentage (*e.g.* Figure 5 of the main text) closely represents the actual doping percentage of the NC cores.

SI-1 – EDX on Yb:YLF Core NCs

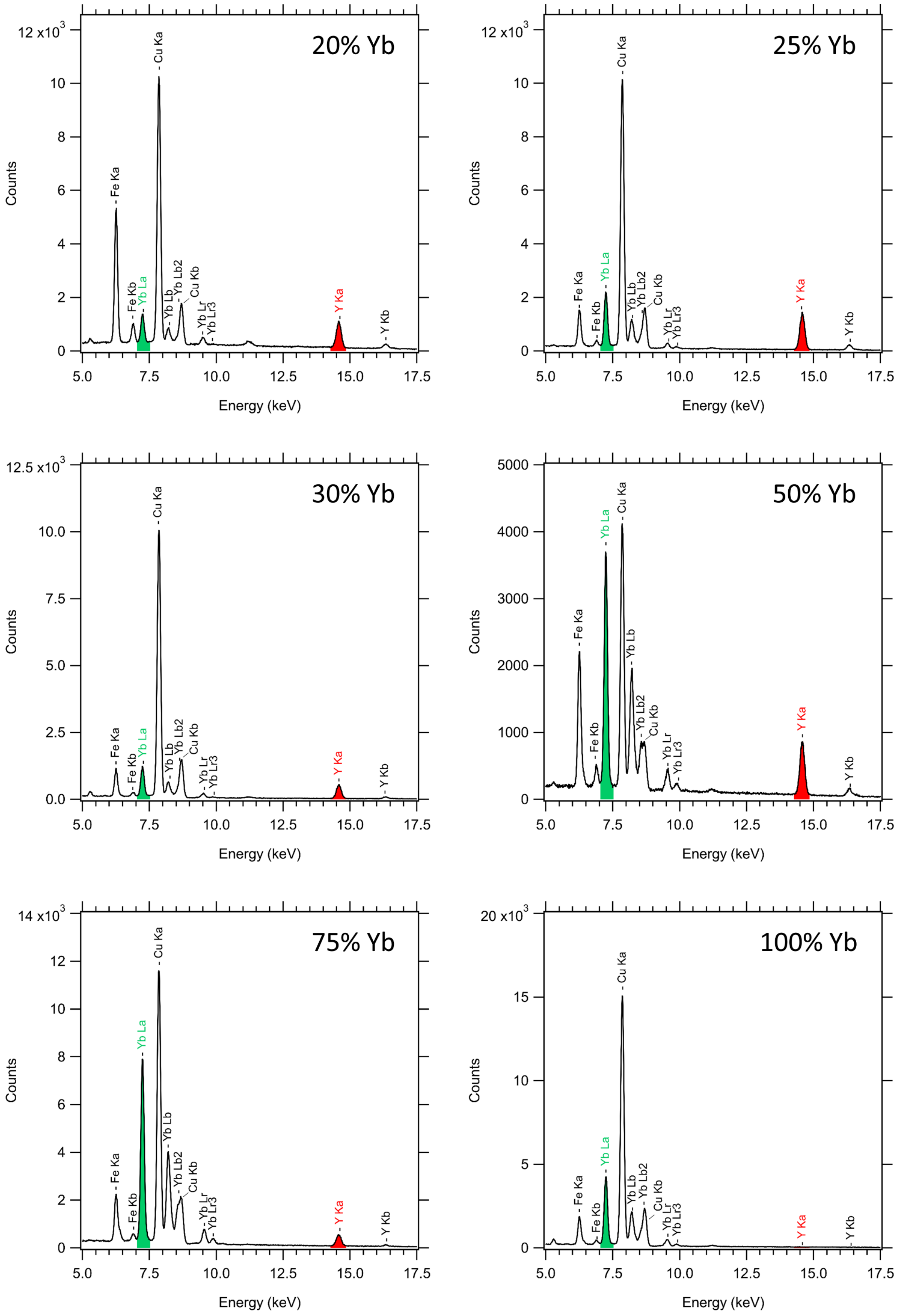

Figure S2. EDX measurements performed on the core NCs (doping concentration 20-100 %) indicate that the incorporation of Y (red) and Yb ions (green) is the same as the intended, weighed-out ratio. Therefore, the Yb core doping percentage (*e.g.* Figure 5 of the main text) represents the actual doping percentage of the NC cores.

SI-2 – XRD of $LiYF_4$, Yb(50%):$LiYF_4$ and $LiYbF_4$ Core NCs

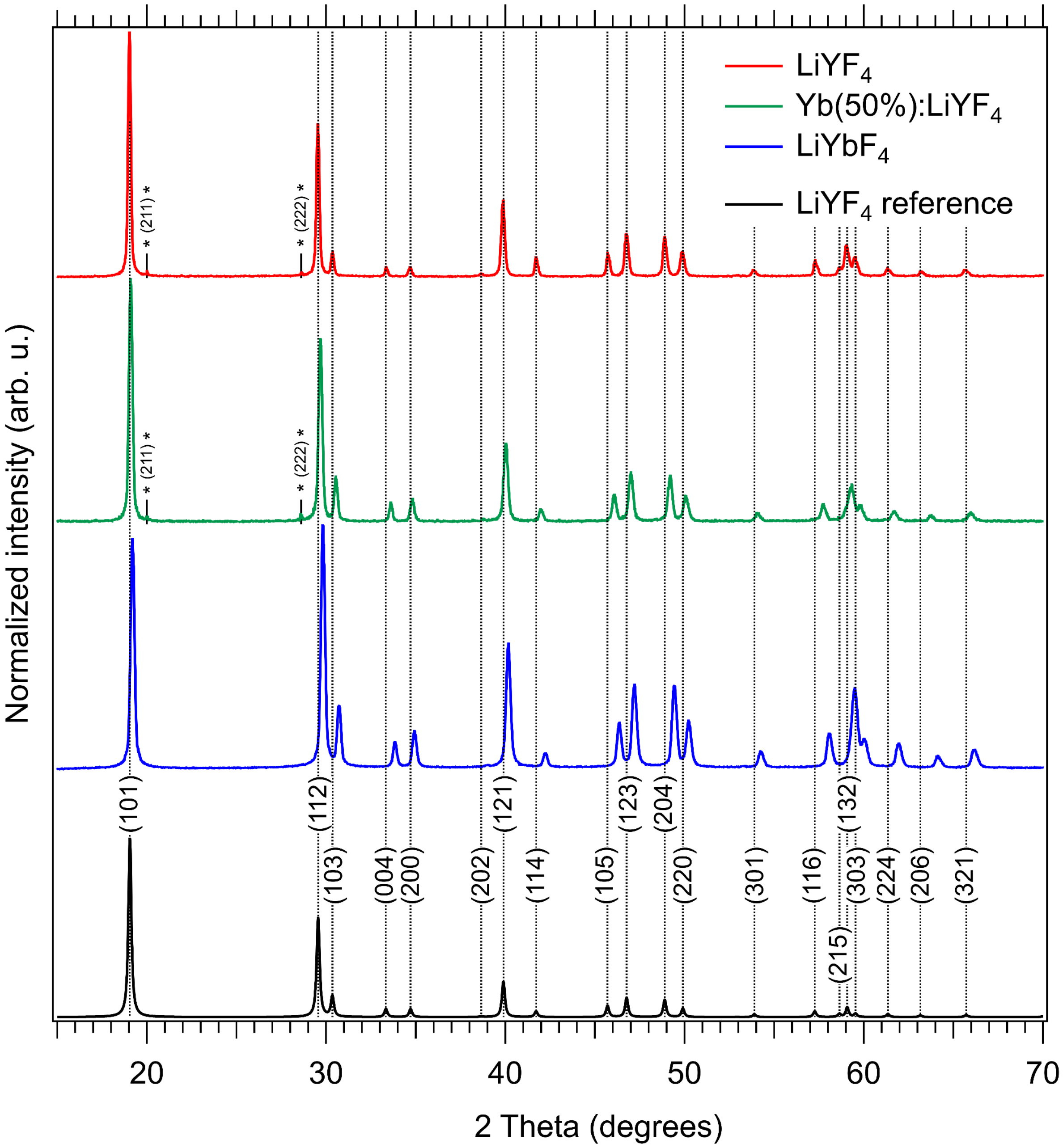

Figure S3. XRD diffractograms of $LiYF_4$, Yb(50%):$YLiF_4$ and $LiYbF_4$ NC cores. The shift to higher angles for higher Yb-fractions indicates a slight lattice contraction. The diffractograms of all samples correspond to the expected scheelite crystal structure without the presence of large, unattributable peaks. The NCs are therefore phase-pure. Small and sharp signals of the (211) and (222) reflections of $Y(b)_2O_3$ are present in the $LiYF_4$ and Yb(50%):$LiYF_4$ samples, indicating the presence of a minute amount of crystalline oxide.

Added as reference is a diffraction pattern of $LiYF_4$ (COD-2001129) reported by Garcia *et al.*[1]

SI-3 – Crystallinity of Yb(25%):YLF/YLF Core/Shell NCs

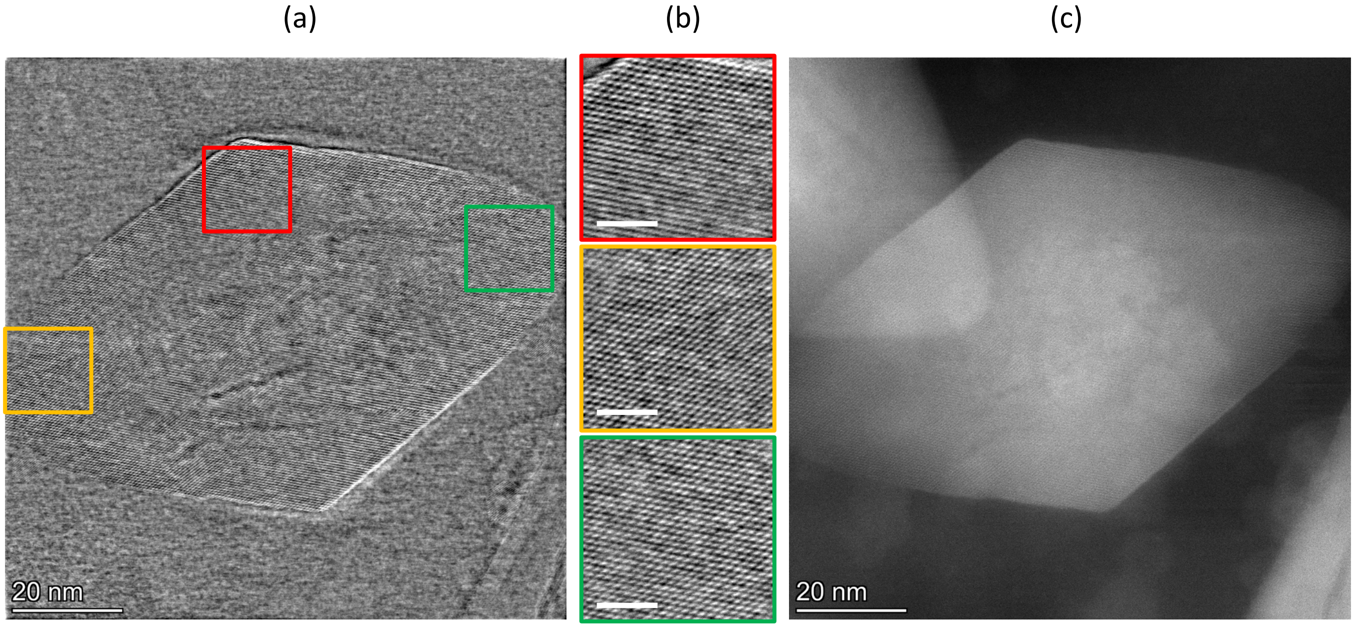

Figure S4. (a) To confirm that the NCs are highly crystalline and do not show a noticeable core/shell lattice mismatch, a core/shell NC was imaged using iDPC and (c) STEM. The image, as well as the constructed diffractogram (Figure S4) from the three indicated areas (a, b) reveal that the NC has a continuous atomic lattice throughout the entire NC, both core and shell (scale bar in (b) corresponds to 5 nm).

Integrated differential phase contrast (iDPC) imaging was used in combination with conventional HAADF STEM imaging to acquire atomic resolution information. It is possible to achieve atomic resolution images with HAADF STEM acquisition alone (Figure S4c), however the electron beam sensitivity of these NCs made it challenging to acquire atomic resolution detail at a sufficiently low magnification as to see the entire NC within a single image. Using a significantly reduced screen current (20 nA), the NC could survive a single atomic resolution image using both HAADF STEM and iDPC imaging. Although the atomic resolution HAADF STEM image is noisy and difficult to interpret, the corresponding iDPC image (Figure S4a, b) clearly shows a single crystalline NC shell.

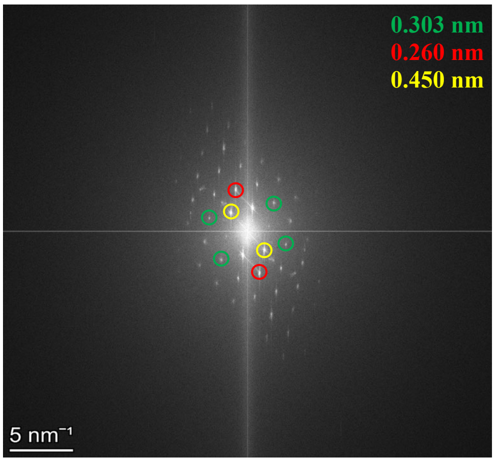

Figure S5. A constructed diffractogram from the three indicated areas of the iDPC image (Figure S4a, b), showing a single crystalline core/shell NC.

SI-4 – Index Matching of NCs with Solvent

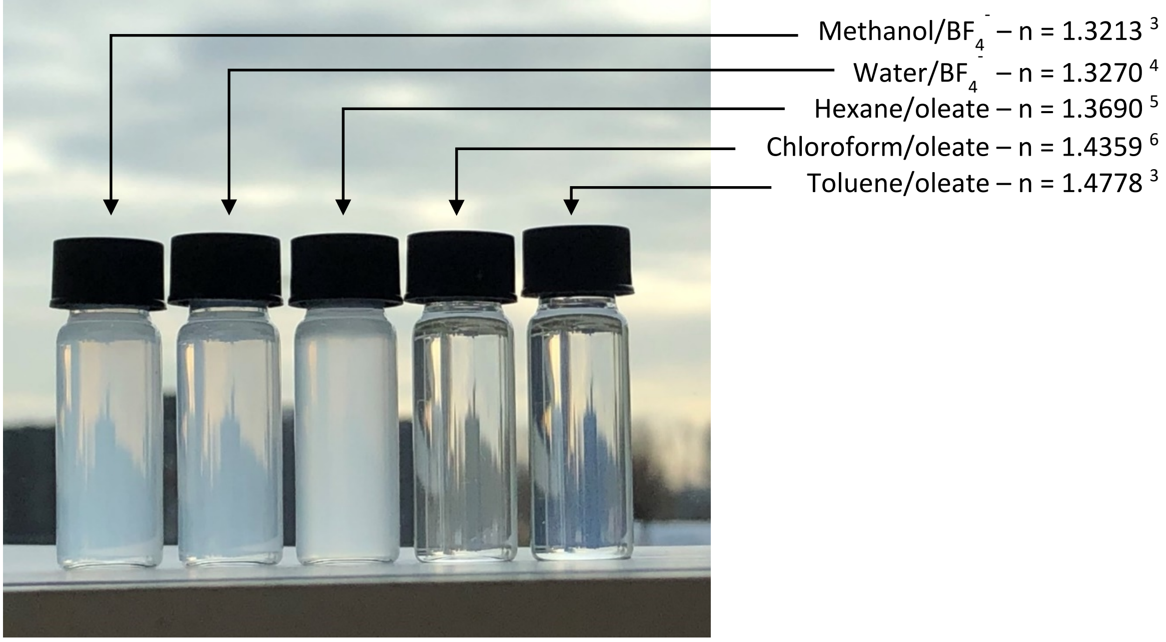

Figure S6. NCs dispersed in different solvents with different refractive indices and surface ligands, showcasing both the improved dispersability with BF_4^- counter ions (as reported by Dong *et al.*[2]) and the significant reduction of scattering due to index matching of the solvent with the NCs. As explained in the main text, using an index-matched solvent removes the correction factor(s) for different refractive indices (and the NC size and shape), resulting in a more accurate PLQY-analysis based on TRPL-spectra. The indicated values for n are used in the determination of the PLQY from the TRPL-model (Equation 1 of main text).[3-6]

SI-5 – Influence of Reabsorption

For all PLQY measurements conducted it has to be ensured that there is no (significant) reabsorption. Reabsorption of the by NCs emitted light will result in a prolonged average lifetime[7] and hence an incorrect increase of the estimated PLQY when using TRPL. For integrating sphere measurements, the PLQY is lowered with increasing reabsorption, assuming the PLQY is not unity.

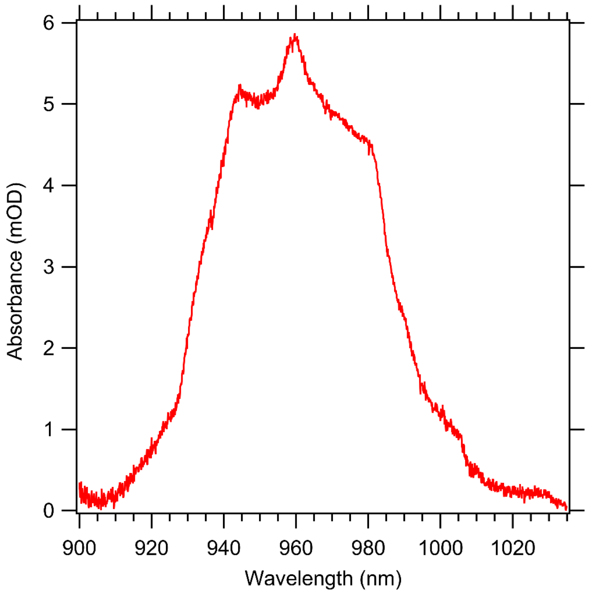

Figure S7. The absorbance spectrum of a Yb(50%):YLF/YLF NC sample in chloroform, used in the main text.

The maximum absorbance is <6 mOD. The transmittance is calculated by:

$T = 10^{-A} = 10^{-0.006} = 0.986$ at the absorbance peak wavelength.

The fraction of reabsorption at the main absorbance peak (*i.e.* not the average absorbance) is thus roughly 1.4%. Reabsorption thus does not play a large role and hence does not lead to a significant increase in PL decay time compared to the intrinsic bulk PL decay time. Therefore, there is no need to correct for reabsorption, as had to be done in *e.g.* the bulk lifetime-analysis reported by Püschel *et al.*[8] Note that for all samples, it was ensured that the overall absorbance was lower than 2% ($A \leq 8.8$ mOD).

SI-6 – PLQY Correction for Reabsorption

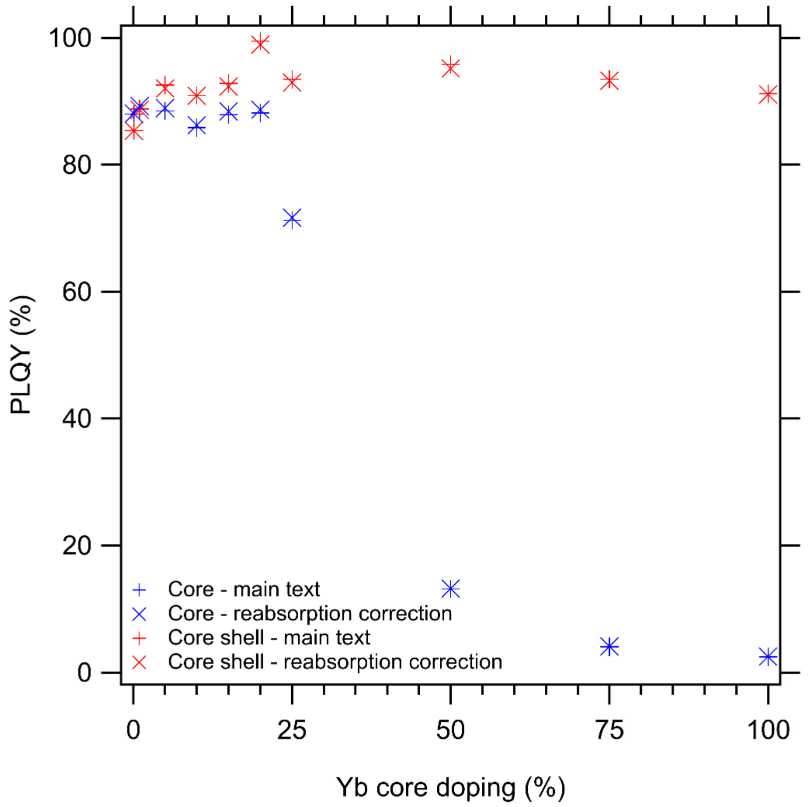

Figure S8. PLQY of the samples shown in Figure 5 of the main text, corrected for any reabsorption of the samples.

To confirm that reabsorption has no influence, we have corrected the reported PLQY (Figure 5a) for reabsorption. We used the following formula from Auzel *et al.*[7] to determine the corrected lifetime (τ_t) from the measured lifetime (τ_i) and a factor for reabsorption (σNl) (Equation S1). We rewrite σNl as a function of the transmittance (T), and obtain the transmittance from, in this case, the PLQY (φ) measurement shown in SI-10, and the integrated counts of the TRPL spectrum of this measurement (I_{tot}). From this, we determined the value for constant k, and subsequently used this constant, together with the I_{tot} and the φ from the TRPL measurements to determine T (Equation S6).

With the transmittance obtained for each sample, we calculated the difference between the measured lifetime (τ_i) and the corrected lifetime (τ_t) (Equation S4). The results of this calculation are shown in Figure S8.

$$\tau_t = \tau_i(1 + \sigma N l) \quad \text{(S1)}$$
$$\tau_t = \tau_i(1 + A * ln(10)) \quad \text{(S2)}$$
$$\tau_t = \tau_i(1 + (-\log(T)) * ln(10)) \quad \text{(S3)}$$
$$\tau_t = \tau_i(1 + \log\left(\frac{1}{T}\right) * ln(10)) \quad \text{(S4)}$$

$$I_{tot} = \varphi(1 - T) * k \quad \text{(S5)}$$
$$1 - \frac{I_{tot}}{\varphi * k} = T \quad \text{(S6)}$$

$$k = 7.5 * 10^8$$

SI-7 – Excitation and Emission Wavelength Dependencies

To validate the TRPL model for a broad range of analyses, it has to be ensured that the PLQY obtained from TRPL-data is not excitation or emission wavelength dependent. Therefore, lifetime measurements were performed for the three emission peaks (shown in Figure S9a), as well as at the lifetime of the generally used emission wavelength (995 nm) when excited at different wavelengths (Figure S9b). From both figures, we can conclude that there is no influence of excitation or emission wavelengths on the obtained PLQY-values.

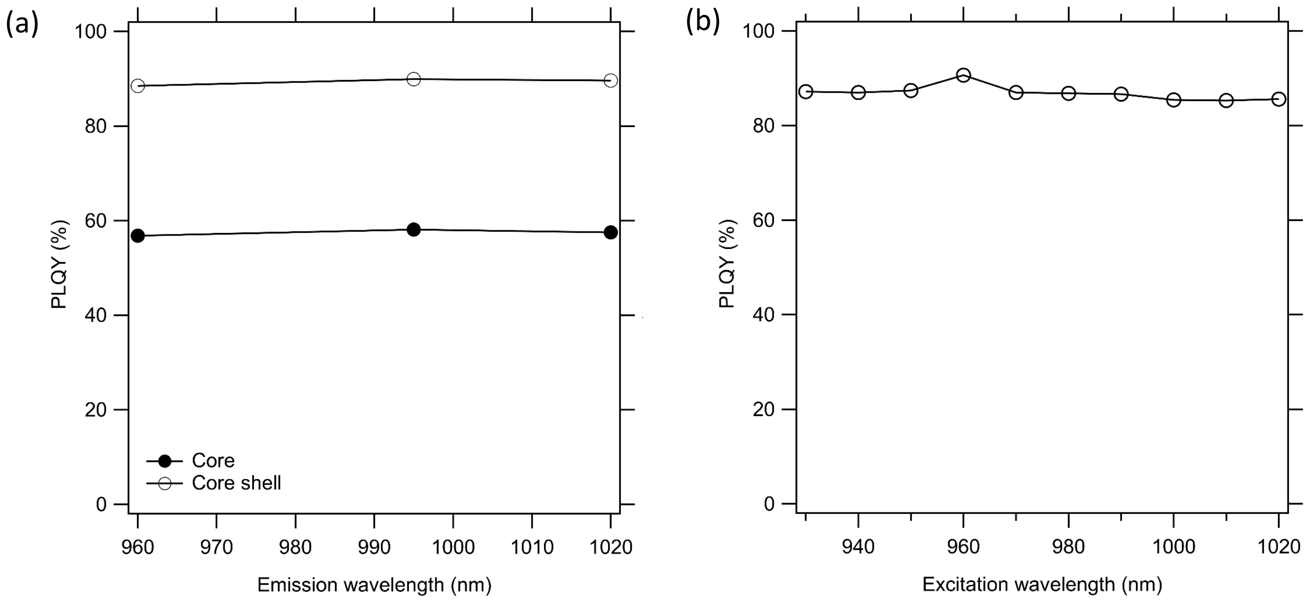

Figure S9. (a) The PLQY extracted for core and core/shell NCs at different emission wavelengths (λ_{ex} = 930 nm), as well as (b) for core/shell NCs at different excitation wavelengths (λ_{em} = 995 nm) show that there is no large dependency of the extracted PLQY to different excitation or emission conditions.

SI-8 – Concentration and Fluence Dependency

In addition to SI-7, we also checked if lowering the excitation fluences and NC concentrations changed any of the obtained PLQY values. As is clear from Figures S10a and S10b, there seems to be no influence on these parameters, again confirming that any reabsorption is not influencing the obtained PLQY values (SI-6).

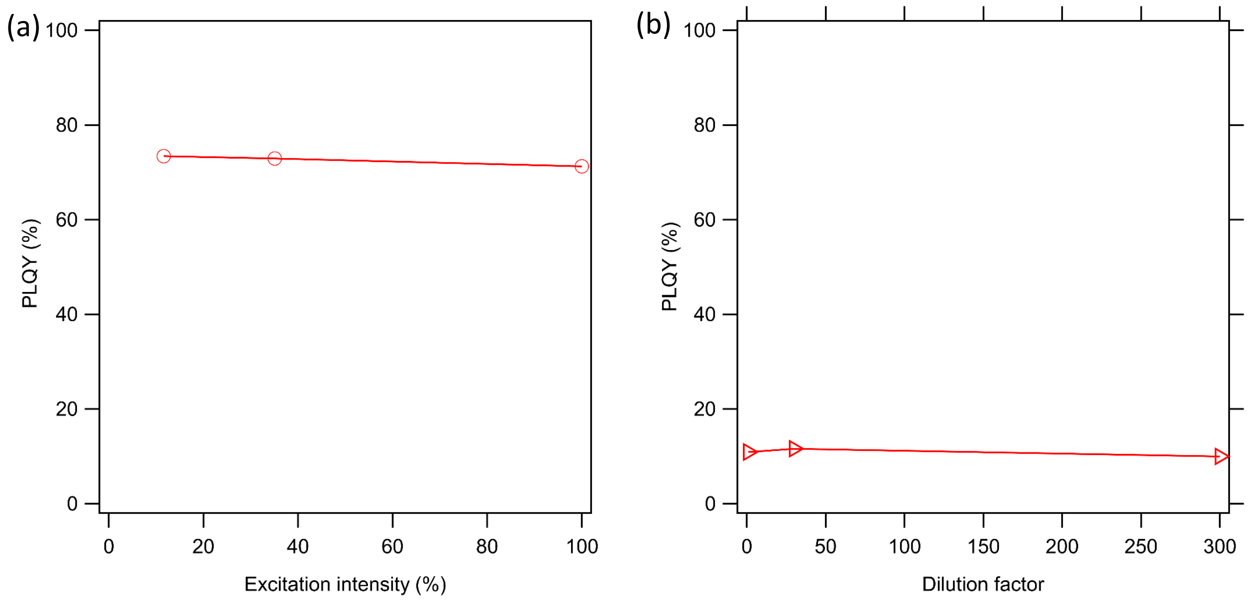

Figure S10. (a) The PLQY extracted for core/shell NCs at different excitation intensities (using gray-filters), as well as (b) for core NCs at different concentrations (by diluting the sample) show that there is no large dependency of the beam intensity or NC concentration. For both figures, λ_{ex} = 930 nm and λ_{em} = 995 nm.

SI-9 – Extended TRPL-Model

The PLQYs of our NC samples have all been calculated using Equation S7 published by Rabouw *et al.*[9]

$$\Gamma_{rad}(n) = \frac{\Gamma_{rad}^{bulk} * n}{n_{NC}} \left(\frac{3n^2}{2n^2 + {n_{NC}}^2}\right) \tag{S7}$$

In this publication Equation S8 is also given, which additionally corrects for the NC size and emission wavelength. However, it is mentioned that the product of the free space wave vector ($k = \frac{2\pi}{\lambda}$) and radius of the spherical(!) particle (a) should be <<1. For the data shown in Figure S11, the ka-value of the non-spherical YLF NCs is roughly 0.45 (a = 90 nm).

$$\Gamma_{rad}(n) = \frac{\Gamma_{rad}^{bulk} * n}{n_{NC}} \left(\frac{3n^2}{2n^2 + {n_{NC}}^2}\right) \left[1 + \frac{(n_{NC}^2 - n^2)(n_{NC}^2 + 10n^2)}{5(2n^2 + n_{NC}^2)}(ka)^2\right] \tag{S8}$$

The changes in the eventual outcome are relatively small for these samples, and due to our NCs being non-spherical and essentially too large for this correction factor, in the main text we have solely used Equation S7. The effect of Equation S8 is shown in Figure S11a and b for NCs dispersed in chloroform and methanol respectively. These figures show that the correction-factor will go to 1 for index-matched solvents, effectively removing the need of the correction.

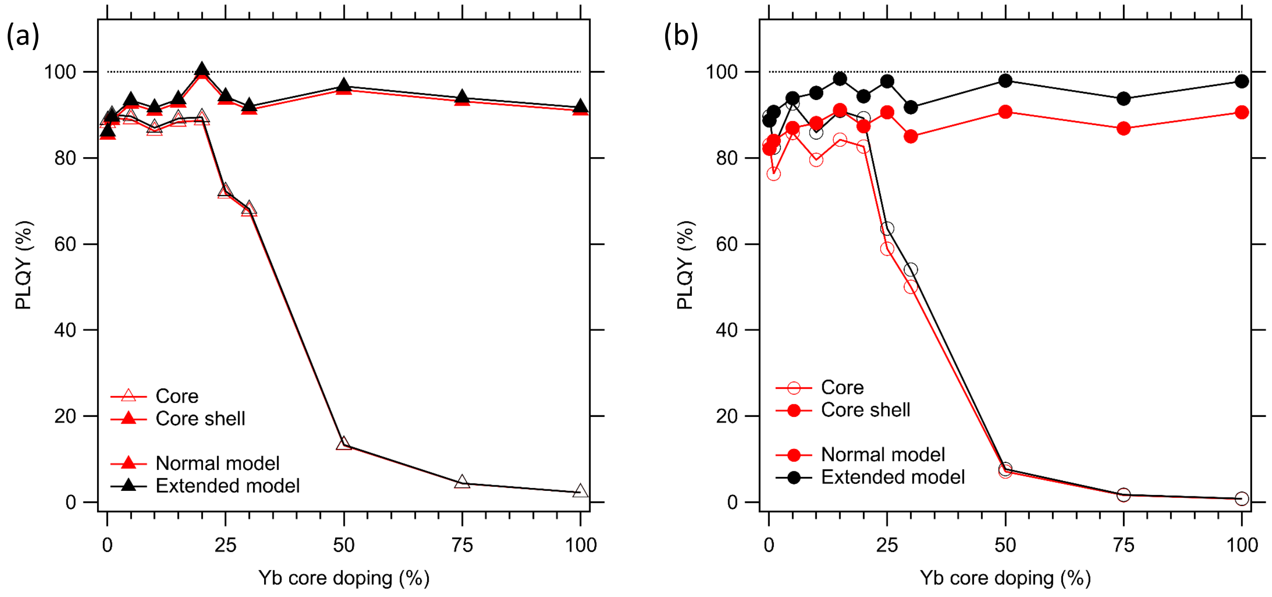

Figure S11. (a) PLQY values for core and core/shell NCs using the "normal" model that is used in the main text, and the extended model described above in chloroform and (b) methanol. For index-matched solvents (*i.e.* chloroform), the correction factor goes to 1 (similar to Equation S7), reducing the error of the analysis.

SI-10 – PLQY from Integrating Sphere (Absorbance)

Another method to estimate the PLQY is using an absorption photospectrometer equipped with an integrating sphere. Here we use a Perkin-Elmer lambda 1050 UV-VIS-NIR photospectrometer, where we compare the measured absorption spectra of samples placed either inside, or well before the integrating sphere.

For the sample inside the sphere, emitted photons will be collected on the detector and will decrease the apparent absorption. For a small Stokes shift between absorbance and emission, the wavelength of an absorbed an emitted photon is nearly the same and so will be the sensitivity of the detector and the transmission of the integrating sphere. This implies that if the PLQY is 100% no absorption appears to take place. When the same sample is measured outside of the integrating sphere (far enough from the entrance so that the fraction of emitted light collected by the sphere is negligible) the detected transmitted light does not contain emitted photons. It is furthermore important to either have a non-scattering sample (index-matched solvent or small enough NCs), or to correct for scattering. In the measurements below, the sample was dispersed in chloroform, and hence practically index matched. From these two absorbance spectra, an estimate of the PLQY can be given, according to:

Sample placed outside of the sphere:

$$A_{out} = -\log\left(\frac{I_0 - I_0 * F_A}{I_0}\right) = -\log(1 - F_A) \quad \text{(S9)}$$

$$\rightarrow \quad 10^{-A_{out}} = 1 - F_A$$

(where A = absorbance, I_0 = incident intensity, F_A = fraction of absorbed light)

Sample inside the sphere (when writing A here, it is understood that this is the *apparent* absorbance, not the true absorbance):

$$A_{in} = -\log\left(\frac{I_0 - I_0 * F_A + I_0 * F_A * PLQY}{I_0}\right) = -\log(1 - F_A + F_A * PLQY) \quad \text{(S10)}$$

$$\rightarrow \quad 10^{-A_{in}} = 1 - F_A + F_A * PLQY$$

$$\frac{10^{-A_{in}} - 10^{-A_{out}}}{1 - 10^{-A_{out}}} = \frac{F_A * PLQY}{F_A} = PLQY \quad \text{(S11)}$$

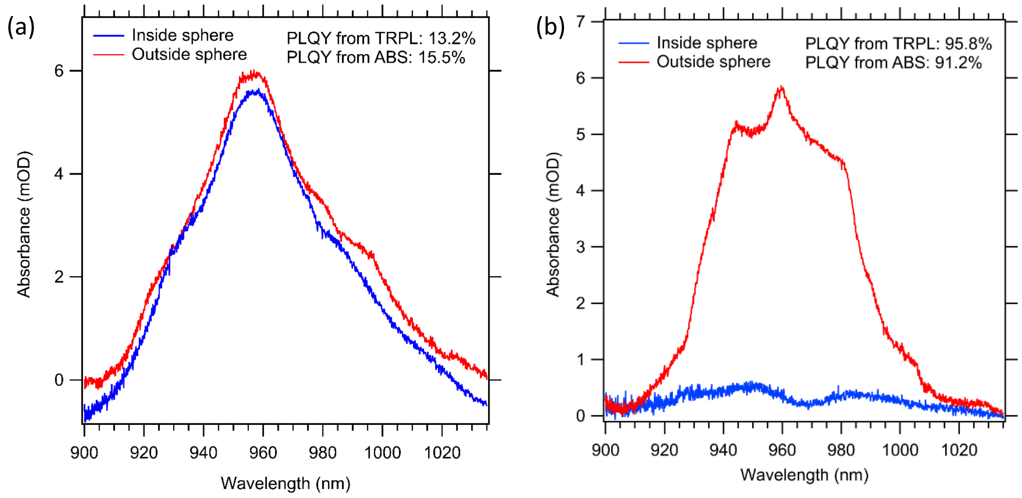

Figure S12. (a) The absorbance spectra of a Yb(50%):YLF core and (b) Yb(50%):YLF/YLF core/shell NC sample in chloroform inside (blue) and outside (red) of an integrating sphere.

Again, the difference of this measurement is relatively small compared to the obtained PLQY from TRPL, confirming that the TRPL-model is adequate at determining the PLQY of Yb:YLF NC samples.

SI-11 – Syntheses With Water – TEM and ED Analysis of Samples

Electron diffraction (ED) analysis (Figure S13a) of the samples synthesized with water added before or after the degassing step reveal that the samples do not significantly change in crystal structure (within the error margin of the measurement). However, TEM images clearly show that the growth of both samples is significantly affected by the presence of (residual) water. Both the sample where water is added after the degassing step (Figure S13b) and before (Figure S13c) show the presence of small particles, next to the expected bipyramidal NCs, indicating that the OH^- likely acts as a growth controlling ligand. For the sample shown in Figure S13b, two PL-lifetimes were found, likely related to the smaller and larger particles. The absence of a biexponential PL-decay for the sample shown in Figure S13c is because the concentration of the smaller particles is far less. Therefore the PL-intensity of these small particles is negligible compared to the large ones. Figure S13d-f shows the EDs of (d) a general YLF sample, (e) the sample that is not degassed after adding water and (f) the sample that has been degassed prior to starting the synthesis.

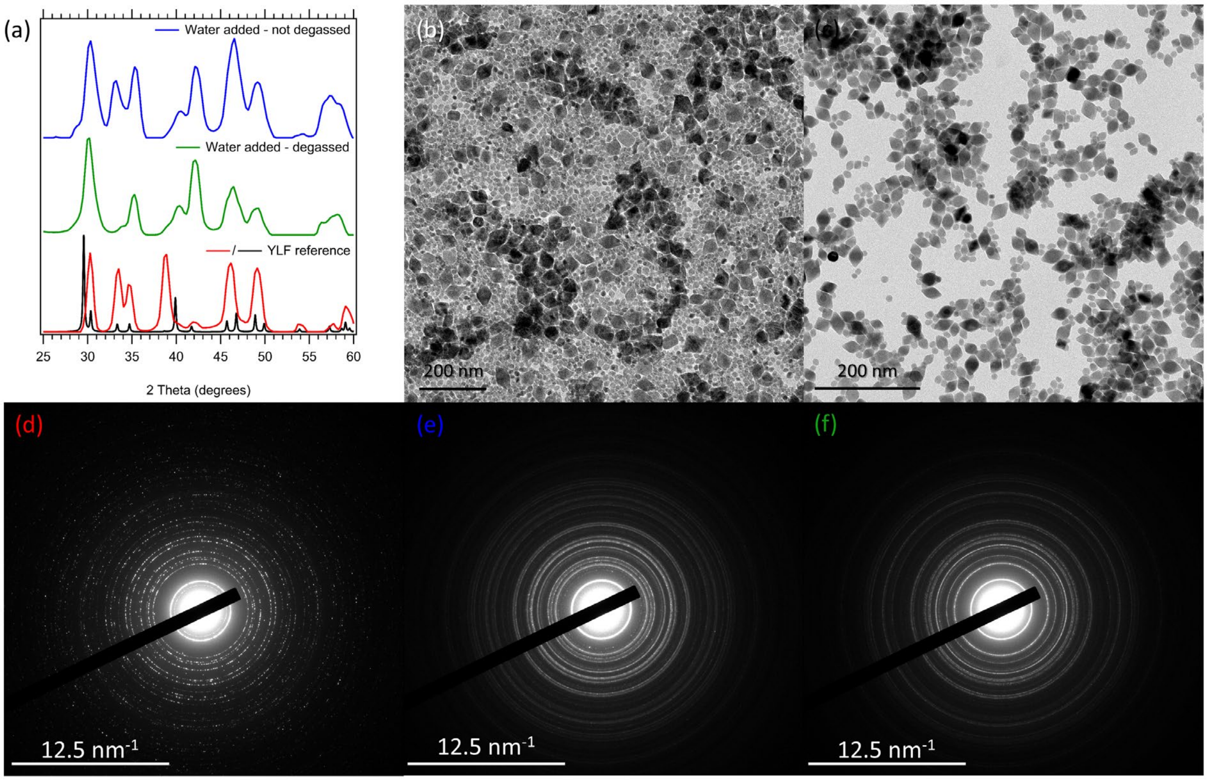

Figure S13. (a) Extracted ED profiles from the raw EDs, shown in Figure S13d-f. (b) TEM image of the sample where, after adding water to the precursor mixture, no degassing step was performed before the synthesis was started. (c) TEM image of a sample where after adding water, the water was removed again by degassing under vacuum. (d) The ED of a general YLF sample, and (e) the ED of the sample shown in Figure S13b, and (f) Figure S13c.

SI-12 – EDX of Yb(25%):YLF/YLF Core/Shell NCs – Lower Magnification

A lower magnification EDX mapping of the sample shown in Figure 4a-c indicates the uniformity of the samples. Furthermore added is the fluoride signal (yellow), showcasing the location of fluoride ions is throughout the entire NC, as expected.

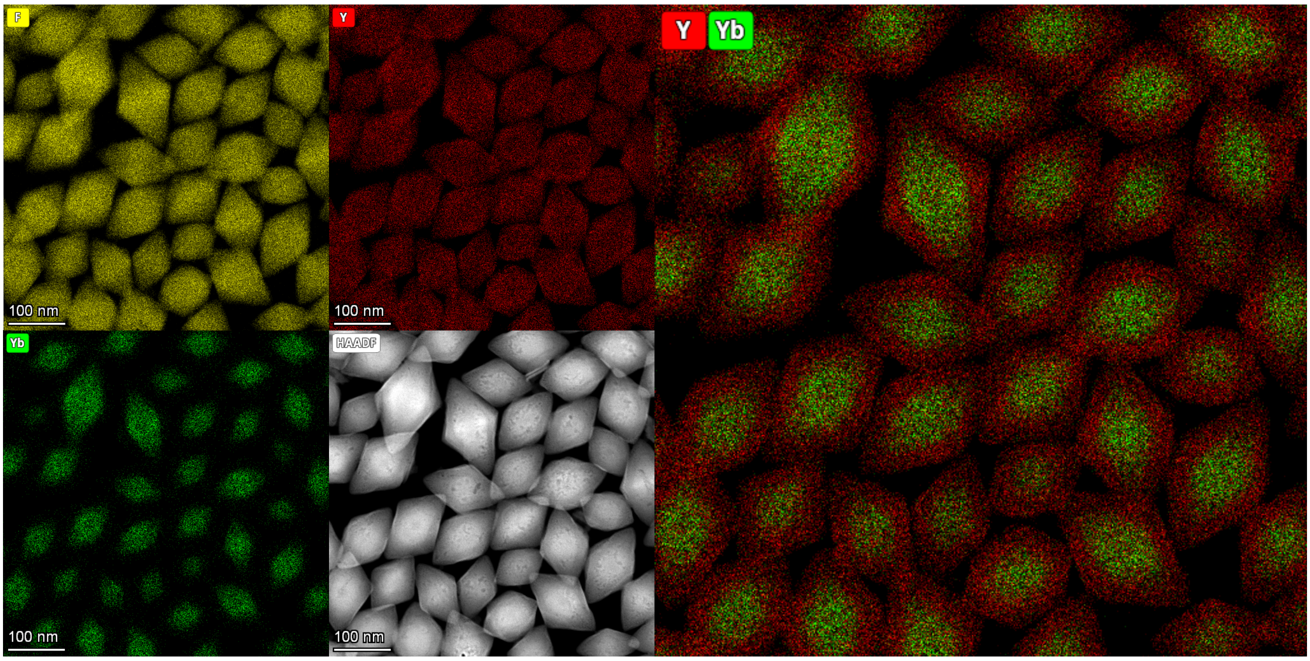

Figure S14. Lower magnification EDX mapping (compared to Figure 4) of Yb(25%):YLF/YLF core/shell NCs confirms the uniformity of the sample. The elemental distribution shown in Figure 4 is thus representative for the sample in general.

SI-13 – EELS Analysis of Yb(25%):YLF/YLF Core/Shell NCs

Apart from using EDX to image the elemental composition, EELS (electron energy loss spectroscopy) was also applied on the same sample as shown in Figure 4a-c and Figure S14. Figure S15a shows again that the location of Yb is mostly isolated to the NC core. Most of the Y signal is found in the shell. Figure S15b indicates that some Yb signal is found in the shell, similar to Figure 4b. An EELS elemental linescan was performed over the NC, as indicated in Figure S15c, and the results shown in Figure S14d indicate again that practically all Yb-signal is found in the core only. Figure S15e shows the full EELS spectrum, with the location of the Y and Yb-signals indicated.

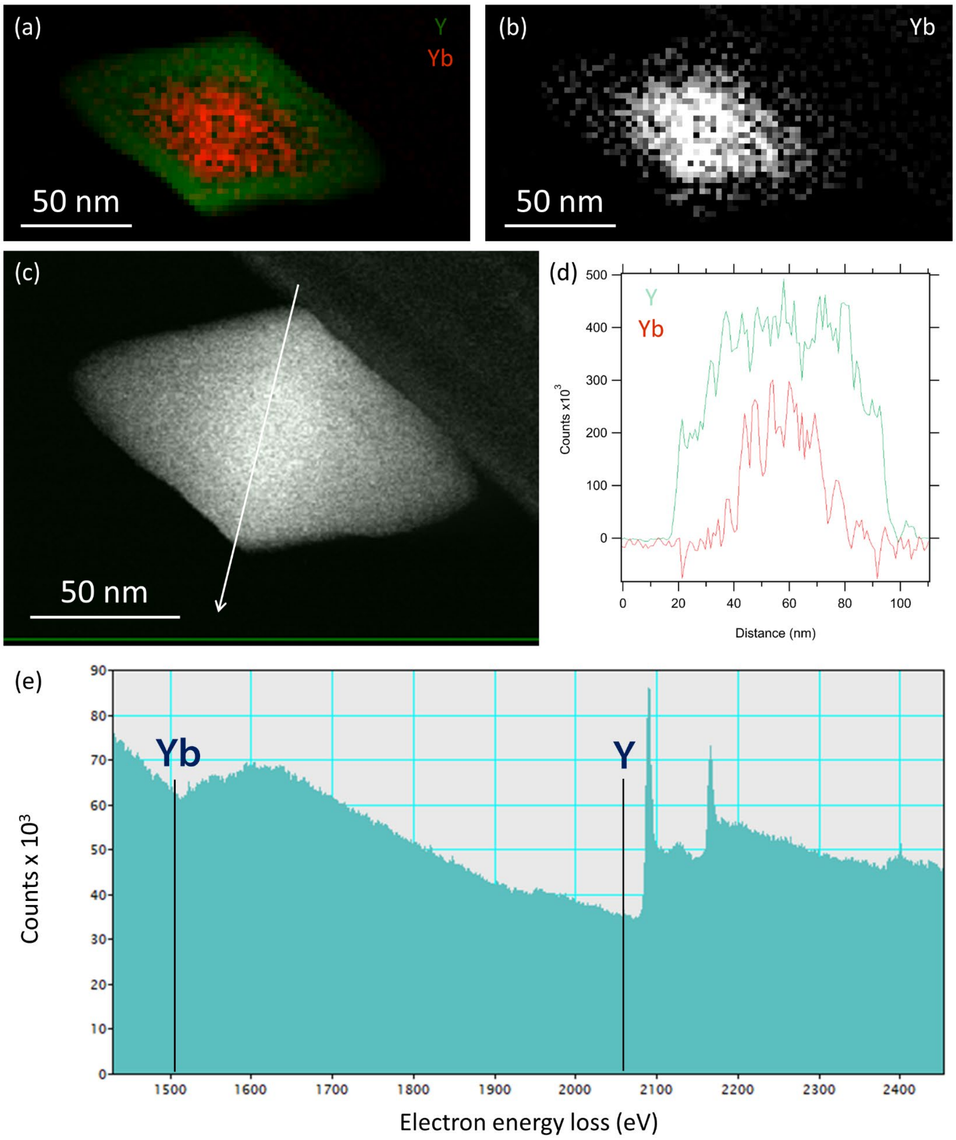

Figure S15. (a) The by EELS measured locations of Y and Yb, as well as (b) solely Yb indicate that most of the Yb-ions remained in the core NC. (c, d) An EELS-linescan over the NC shows that indeed the Yb-signal in the shell is at the level of the background noise. (e) The full EELS-spectrum, used for the determination of the locations of Y and Yb in the NC shown.

SI-14 – Förster Resonance- and Dexter-Type Energy Transfer Simulations

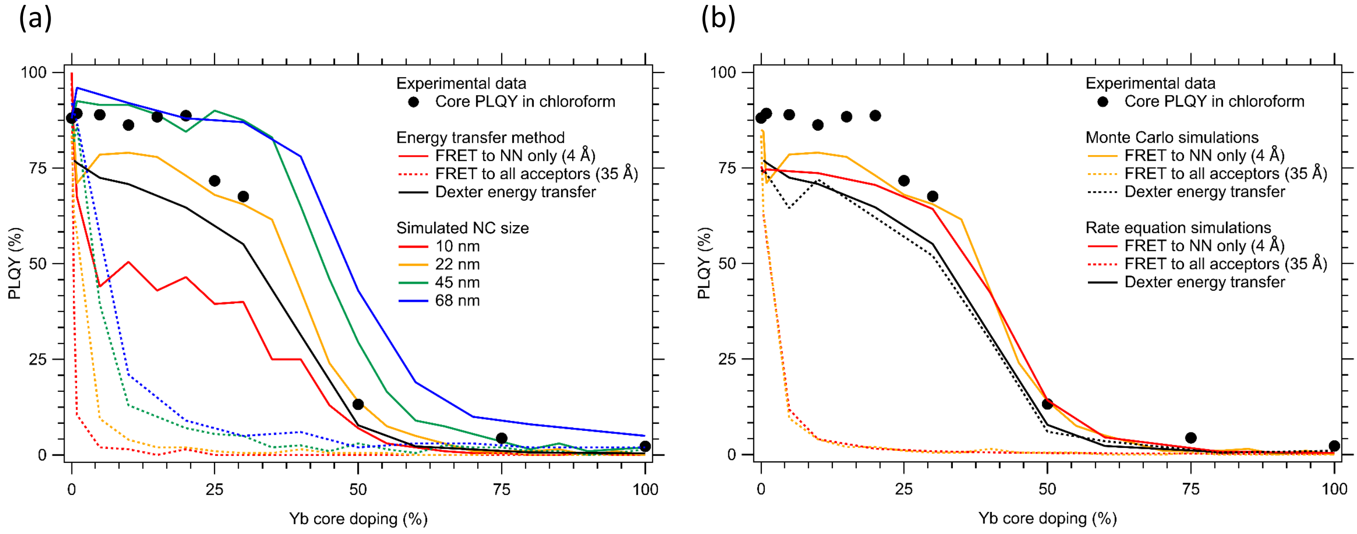

Figure S16. (a) PLQYs derived from Monte Carlo simulations modelling FRET or Dexter-type energy transfer. The models used different NC sizes, as well as a differing FRET-search radius, including either all acceptors or only nearest neighbors (NN). (b) A comparison between Monte Carlo simulations and rate-equation based simulations for the 22x15x15 nm NC.

Modelling FRET in Yb:YLF

FRET from excited Yb atoms to the NC surface was modelled in two ways: Monte Carlo simulations, and rate equation calculations. Both methods make use of a NC model that is cut from the YLF bulk,[1] with the NanoCrystal tool,[10] exposing only (101) facets. The data shown in the main text is based on a 22x15x15 nm NC. The Monte Carlo simulations shown here were also carried out for NC models of different sizes (as summarized in Table S1). For computational efficiency, the Li and F atoms are not taken into account, leaving only the Y atoms. At the start of each simulation, part of the Y atoms (the total amount depending on the doping concentration) is randomly changed to Yb. As only the Yb are relevant for modelling FRET, the remaining Y atoms are then discarded.

Table S1. General parameters of the different modeled NCs for the Monte Carlo simulations.

NC name	Atoms	Surface atoms	Dimensions (nm)	Used in main text
NC_10nm	1,430	686	10x7x7	
NC_22nm	13,706	3,446	22x15x15	Yes
NC_45nm	105,952	9,440	45x30x30	
NC_68nm	353,600	32,006	68x46x46	

Monte Carlo simulations

To determine the quantum yield via Monte Carlo simulations, 200 independent excitations paths (100 for the 68x46x46 nm NC to limit computational costs) are simulated for each doping concentration. At t=0, 200 random Yb sites are selected as “excited”. If the number of Yb atoms is lower than 200 (which may happen for small NCs at low doping concentrations), the same Yb atom can be selected multiple times. Next, for each “excited” Yb atom, the distance to all other Yb atoms (excited or not) in a radius of R_{search} is computed. For “normal” FRET,

where the excitation can be transferred to any nearby Yb atom, $R_{search} = 3.5\ nm$. For the simulations where FRET transfer is restricted to nearest neighbors only, $R_{search} = 0.4\ nm$. Based on the distance to the surrounding Yb atoms, the FRET rate is computed to each of these Yb sites, using $k_{FRET} = k_{rad} \times \left(\frac{R_0}{R_{Yb}}\right)^6$, where $k_{rad} = 4.55 \times 10^{-4}\ \mu s^{-1}$, $R_0 = 1.5\ nm$, and R_{Yb} the distance to the other Yb atoms. The probability of FRET to each atom is then determined with $P_{FRET} = k_{FRET} \times dt$, with $dt = 0.04\ \mu s$. The probability of radiative decay is given by $P_{rad} = k_{rad} \times dt$.

To determine the location of the "excitations" at the next time step, a randomly generated number then determines which event takes place (the probability that nothing happens given by $P_{nothing} = 1 - P_{rad} - \sum P_{FRET}$). The "excitations" are propagated in this way for a maximum of 20,000 μs. If an excitation reaches a surface atom within the time limit of the simulation, it is removed and no longer propagated. We define surface atoms as any atom that has ≤ 50 neighbors within a 1 nm radius. Practically, this amounts to the first 1-2 monolayers of the NC surface. At the end of the simulation, the quantum yield is determined through $PL\ QY = \frac{N_{rad}}{200 - N_{act}}$, where N_{rad} is the number of excitations that have decayed radiatively, and N_{act} the number of excitations that have neither decayed nor reached the surface.

Rate equation calculations

The rate equation calculations use the same parameters (*i.e.*, k_{rad}, R_0, dt) as used in the Monte Carlo simulations. However, instead of 200 explicit excitation pathways, all Yb sites now start with 1 "excitation". The radiative decay rate and the FRET rates between all Yb sites are stored in an $(N_{Yb} + 1) \times (N_{Yb} + 1)$ matrix, where N_{Yb} is the number of Yb atoms in the system. In this matrix, element $k_{i,j}$, gives the FRET rate from Yb_i to Yb_j. The last element of each row (*i.e.*, $k_{i,(N_{Yb}+1)}$) gives the radiative decay rate. All the rates *from* Yb sites at the surface are set to 0, to prevent excitations from leaving the surface. For "normal" FRET (*i.e.*, **not** restricted to nearest neighbors), rates smaller than $\frac{k_{rad}}{50}$ are also set to 0 for computational efficiency. For simulations where only transfer to nearest neighbors is allowed, all rates smaller than $1.9\ \mu s^{-1} \approx k_{FRET,nearest-neighbour}$ are set to 0.

The "excitation populations" are then propagated via $N_{t+1} = N_t + (N_t \cdot K_{rate}) \times dt$, where N_t is the "excitation population" of each Yb atom at time=t, and K_{rate} is the matrix with all rates. For doping concentration <10%, only few Yb are present. To reduce the numerical noise, the average quantum yield from 5 independent runs was taken.

Comparison with experimental results

Figure S16a compares the experimentally measured PLQY decay as function of Yb doping concentration, with the results from the Monte Carlo simulations. As also discussed in the main text, simulation of "normal" FRET leads to an immediate decrease in PLQY and hence fails to reproduce the experimentally observed plateau at lower doping concentrations. In contrast, the plateau is obtained when only allowing transfer to nearest neighbors. Figure S16b shows that these results are not dependent on the computation method. Both Monte Carlo simulations and the rate equations methods give the same results for both "normal" and nearest neighbor FRET.

Modelling Dexter-type electron transfer in Yb:YLF

Like FRET, Dexter-type electron transfer (DET) was modelled for the 22x15x15 nm NC, using both Monte Carlo simulations, and rate equations calculations. In both cases, the methods were completely analogous to those described in the previous section for FRET, with the exception that now $k_{Dexter} = A \times \exp(-\beta \times r)$. Here, $\beta = 0.5\, nm^{-1}$. This value is higher than the value of 0.1 nm^{-1} that is more often used. Lower values of β fail to reproduce the PLQY plateau at low Yb^{3+} concentration. This shows that the ET mechanism must have a very steep distance dependence. Since we showed already that FRET to nearest neighbors gives an accurate description of the experimentally observed PLQY curves, the DET rate at the nearest neighbor distance must be the same as the FRET rate in that case. This defines the prefactor $A = 2.38 \times 10^8\, \mu s^{-1}$.

Comparison with experimental results

Figure S16a compares the experimentally measured PLQY decay as function of Yb doping concentration, with the results from the Monte Carlo simulations of FRET. As also discussed in the main text, simulation of "normal" FRET leads to an immediate decrease in PLQY and hence fails to reproduce the experimentally observed plateau at lower doping concentrations. In contrast, the plateau is obtained when only allowing transfer to nearest neighbors.

Such very short-range transfer may be more consistent with Dexter-type electron transfer, where the transfer rate depends exponentially on the distance between the Yb atoms. Indeed, as shown in Figure 5a in the main text, as well as Figure S16a, Dexter-type transfer can accurately describe the plateau at lower doping concentrations. It must be noted that the values of β and A were not fitted to the data, but estimated based on previous research[11] and the ability of the simulation to describe the experimental data. Thus, although the values of β and A may not be exactly correct, it *is* possible to describe the experimental data with a Dexter-type model, whereas we were unable to describe the data with FRET, regardless of the values of the parameters.

Figure S16b shows that these results are not dependent on the computation method. Both Monte Carlo simulations and the rate equations methods give the same results for both "normal" and nearest neighbor FRET, and Dexter-type electron transfer.

SI-15 – PLQY of NCs in Different Solvents

To test the influence of solvents and solvent-related PL-quenching on the obtained PLQY-values, the PLQY of both core and core/shell NCs were measured for a large range of doping fractions. As can be seen from Figure S17a (identical to Figure 5c of the main text) and Figure S17b, all NC samples follow the exact same trend, albeit that the PLQY of the more polar solvents (containing –OH groups) is systematically slightly lower. As explained in the main text, especially for the core-NCs in water we observe a systematically lower PLQY compared to all other solvents, indicating that this is not solely related to the difference in refractive index. However, as the PLQY values obtained are not significantly different, and the trends are the same for all particles, we conclude that the influence of the solvent on the PLQY is not negligible, but still small compared to other PL-quenching factors that likely reside on the surface of the core NCs.

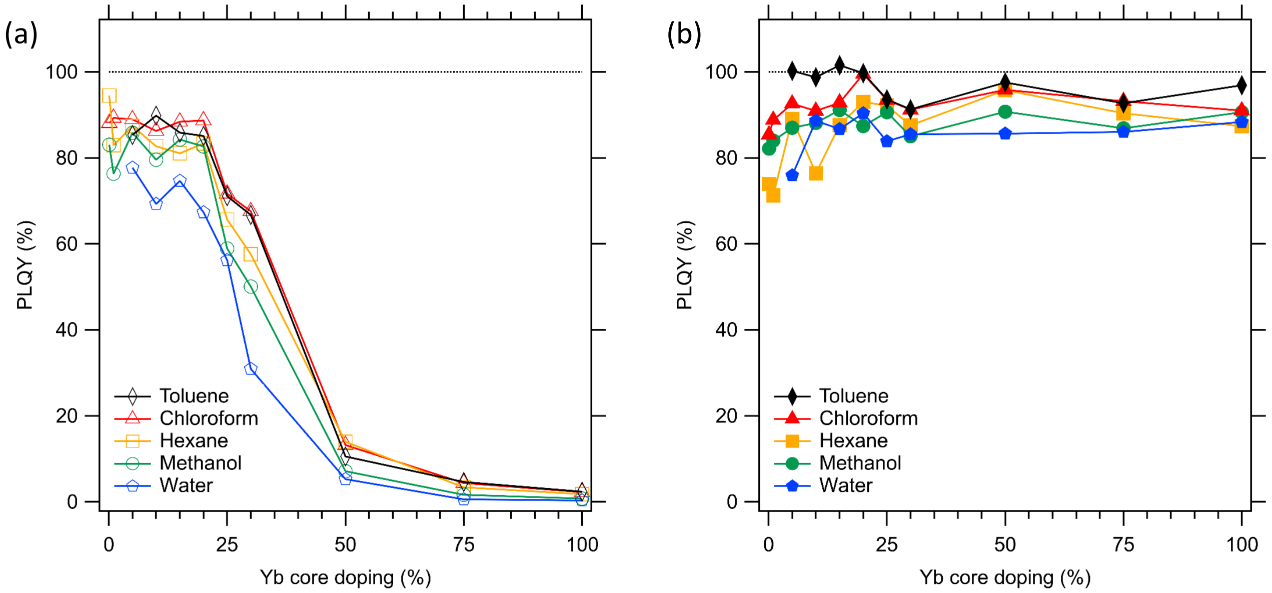

Figure S17. (a) PLQY of Yb:YLF core and (b) core/shell NCs in different solvents. The trends discussed in the main text are true for all different solvents measured. This means that the errors introduced by the differences in refractive indices are relatively small.

SI-16 – Effect of the Refractive Index on the Average PLQY

TRPL-measurements on Yb:YLF NCs showed a systematically lower PLQY for samples in water than for the same samples in chloroform (Figure 5d of main text). As this can either be (1) a solvent quenching effect, indicating that the 15 nm shells are insufficient to mitigate all PL-quenching from core through the shell to the surface or (2) a direct effect of a larger mismatch in refractive index with YLF (n=1.4485) to water (n=1.327) than to chloroform (n=1.4359), apart from measuring $LiYbF_4$ NCs with a 15 nm or 30 nm thick shell (showcasing no difference in PLQY and hence making (1) unlikely), we also measured the PLQY of the 30 nm thick core shell shell NCs in all other solvents, and averaged the PLQY for samples with a low doping density (0.1-15 %) and samples with a high doping density (75 and 100%), and observed the PLQY trends for core and core shell NCs, as shown in Figure S18.
Figure S18a shows the average PLQY for core and core shell NCs with low doping densities (0.1-15% Yb), and showcases a roughly linear trend of increasing PLQY with increasing refractive index. The same is trend can be observed for high Yb-doped (b) cores, and (c) core shell and core shell shell (only 100% $LiYbF_4$-core based) NCs. Furthermore, from Figure S18c it is clear that the 30 nm thick shells are not better than the 15 nm thick shells, showcasing that the 15 nm thick shell is sufficient to mitigate any energy transfer from core to the NC surface. It has to be noted that the core samples in water systematically have lower PLQY.

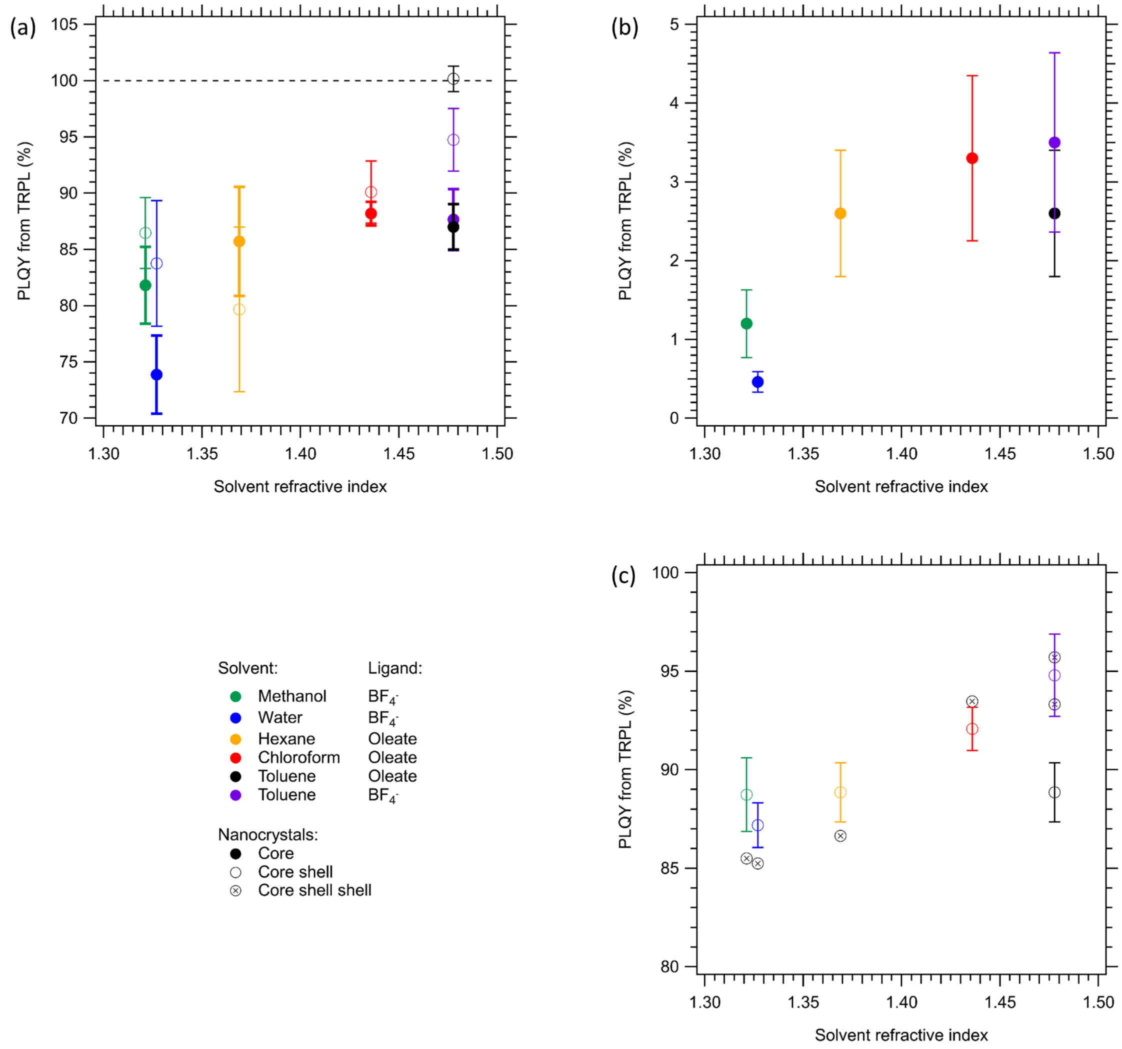

Figure S18. (a) Average PLQY for low and (b) high doped core and (c) core shell NCs. All three figures show a roughly linear increase of PLQY with increasing refractive index.

SI-17 – PLQY for Optical Refrigeration

A quick calculation shows what the PLQY of the NC sample should be, in order to optically refrigerate (effective phonon absorption), instead of heat (effective phonon emission). Note that the average emission energy ($\overline{E_{em}}$) depends on the temperature (*i.e.* available phonons) and hence will move to lower energies when cooling down. This results in a different required PLQY to optically refrigerate for any given temperature.

$$Heating = Cooling$$

$$(1-\varphi)E_{ex} = \varphi(\overline{E_{em}} - E_{ex}) \quad \text{(S12)}$$

$$E_{ex} - \varphi E_{ex} = \varphi\overline{E_{em}} - \varphi E_{ex} \quad \text{(S13)}$$

$$\varphi \geq \frac{E_{ex}}{\overline{E_{em}}} \quad \text{(S14)}$$

For $E_{ex} = 1030\ nm\ (E = 1.204\ eV)$ and $\overline{E_{em}} = 992\ nm\ (E = 1.250\ eV)$, $\varphi \geq 96.3\%$

SI-18 – Temperature Dependent Emission Spectra

It is reported that the temperature of Yb:YLF can be derived from the bulk emission spectrum.[12,13] To set a proper reference, the emission of Yb:YLF NCs was obtained at different temperatures ranging between 80K and 200K. It is clear from the spectra in Figure S19a, that the lower wavelength emission peaks get reduced in intensity at lower temperatures, indicating the lower number of phonons in the NCs. Note that these temperatures are applied in a cryostat, and hence not optically induced. (b) The ratio of peak B/A (indicated in Figure S19a) has an almost linear trend, shown in Figure S19b, as well as (c) the average emission wavelength at different temperatures. These trends were used to identify any optical cooling from the emission spectra, as shown in SI-19.

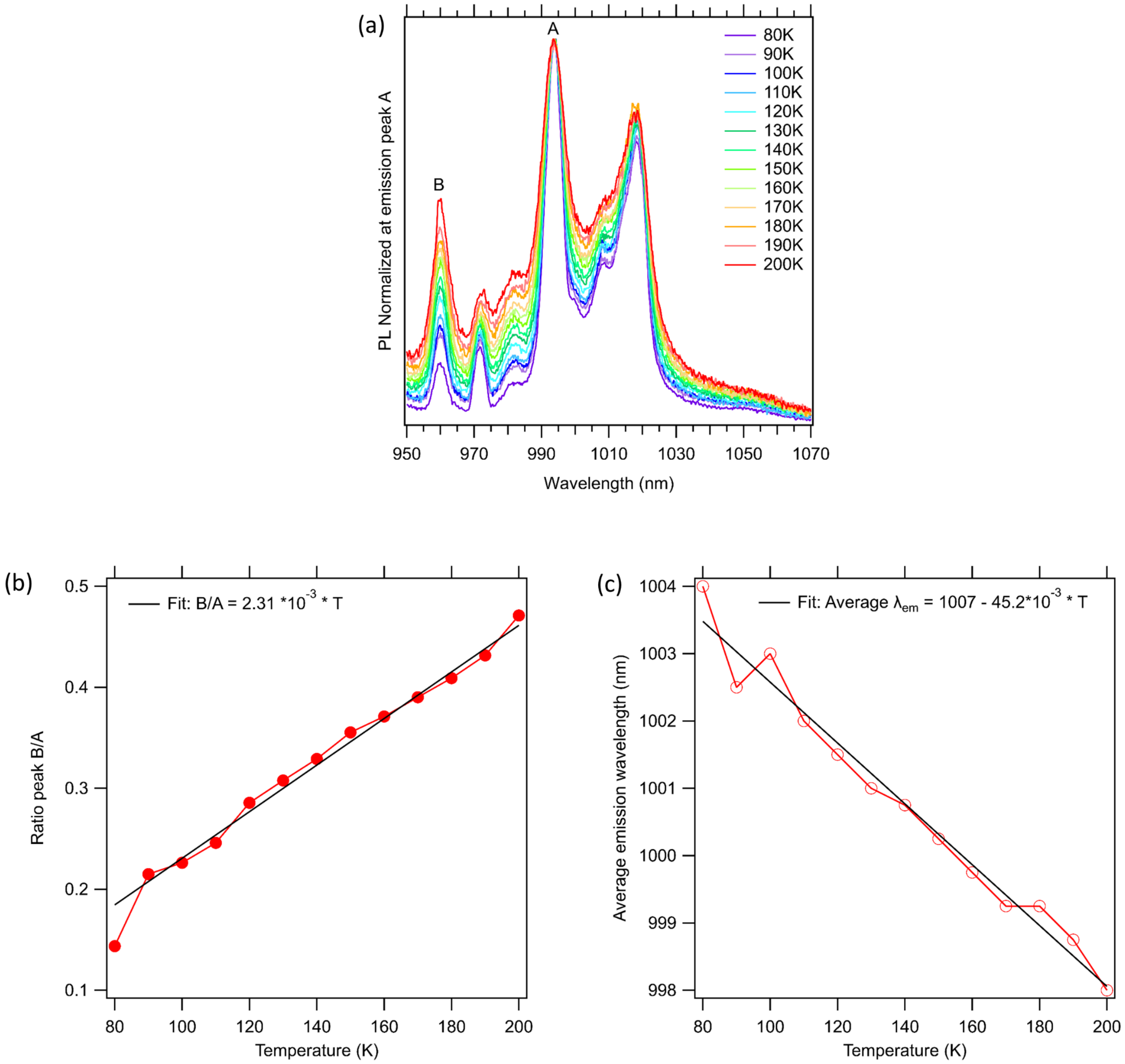

Figure S19. (a) Emission spectra of Yb:YLF NCs at different temperatures (applied by a cryostat) and the trends of (b) emission peaks and (c) average emission wavelength versus temperature. Both (b) and (c) show linear trends that can be used to determine the temperature of the NCs from their emission spectra.

SI-19 – Temperature of NCs from Emission Spectra

As shown in Figure S20, the PL-spectrum of Yb:YLF changes when the temperature changes, due to a lower number of available phonons at lower temperature, resulting in a reduced intensity of higher energy PL-peaks. In reports, this phenomenon is used to conclude that Yb:YLF and Yb:$NaYF_4$ are showing optical refrigeration.[14] However, as shown in Figure S20, a sample with low PLQY (13.2%, Figure S20a) shows practically identical PL-spectra as a sample with a high PLQY (95.8%, Figure S20b) when excited at 930 nm (to increase heating) and 1030 nm (to reduce heating). Similarly, the average emission wavelength (indicated by the straight lines in Figure S19) is similar for both samples.

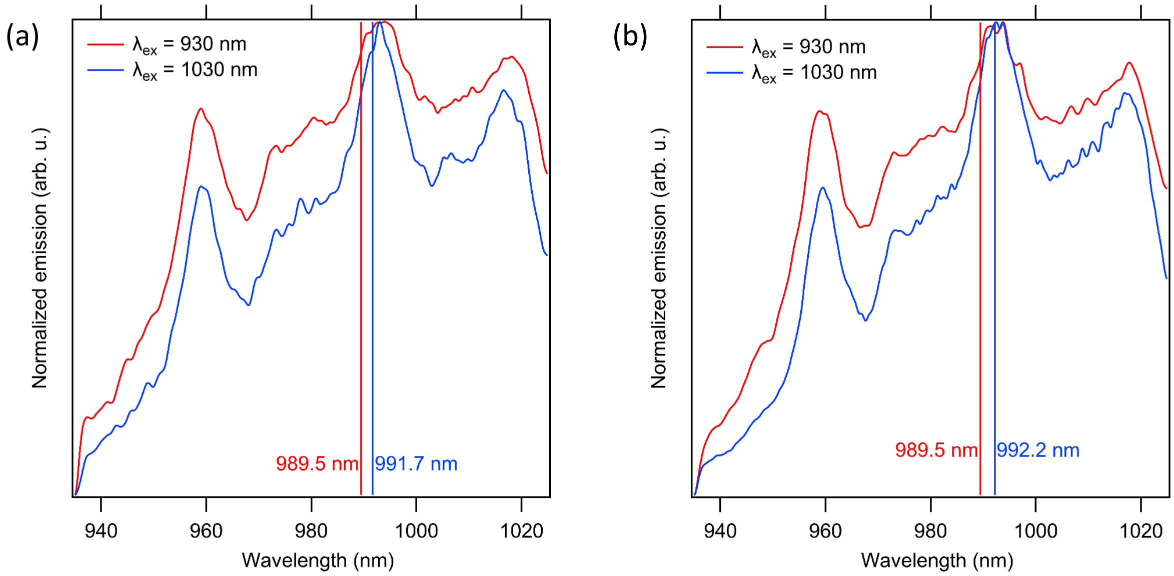

Figure S20. (a) Emission spectra of NCs excited at 930 nm (resulting in phonon-emission for all samples) and 1030 nm (to significantly reduce phonon-emission in high PLQY samples) for a low and (b) a high PLQY sample. As is clear from this image, the large difference in PLQY does not show a significant difference in the average emission, nor in the ratio of peak B/A (see Figure S19a).

The peak ratio B/A for both NC samples is shown in Table S2. From this data we see that the analysis of PL-spectra and the extraction of the NC temperature is per definition not straightforward, and in our case not applicable as indication for the temperature. This, because a low PLQY-sample (that should heat significantly) gives the same temperature and temperature change as a high PLQY sample (that should at least show far less heating than the low PLQY sample). Note that the above shown PL-spectra (Figure S20) are obtained from a NC film on PTFE, to reduce heating of the NCs through the substrate as much as possible. Identical results are obtained when measuring a dispersion in chloroform. Therefore, it is likely that the temperature of the NCs in these measurements is determined by the temperature of the environment, and that the PL-spectra change at different excitation WLs. Only PL-spectra of NCs that are isolated from external heating can therefore be applied for a proper temperature analysis. The analysis of PLQY through TRPL is therefore in all practical cases a much better analysis.

Table S2. The extracted temperatures for two NC samples (high and low PLQY), excited at 930 nm and 1030 nm, calculated using the fitted functions shown in Figures S19b and c.

Excitation wavelength - PLQY	Temperature according to peak ratio (K)	Temperature according to average emission wavelength (K)
930 - Low	354.1	389.4
930 - High	354.1	389.4
1030 - Low	282.7	340.8
1030 - High	282.0	329.7

References

1. Garcia, E.; Ryan, R. R. Structure of the Laser Host Material $LiYF_4$. *Acta Crystallogr. Sect. C Cryst. Struct. Commun.* **1993**, *49* (12), 2053–2054.
2. Dong, A.; Ye, X.; Chen, J.; Kang, Y.; Gordon, T.; Kikkawa, J. M.; Murray, C. B. A Generalized Ligand-Exchange Strategy Enabling Sequential Surface Functionalization of Colloidal Nanocrystals. *J. Am. Chem. Soc.* **2011**, *133* (4), 998–1006.
3. Moutzouris, K.; Papamichael, M.; Betsis, S. C.; Stavrakas, I.; Hloupis, G.; Triantis, D. Refractive, Dispersive and Thermo-Optic Properties of Twelve Organic Solvents in the Visible and near-Infrared. *Appl. Phys. B Lasers Opt.* **2014**, *116* (3), 617–622.
4. Hale, G. M.; Querry, M. R. Optical Constants of Water in the 200-Nm to 200-Mm Wavelength Region. *Appl. Opt.* **1973**, *12* (3), 555.
5. Kozma, I. Z.; Krok, P.; Riedle, E. Direct Measurement of the Group-Velocity Mismatch and Derivation of the Refractive-Index Dispersion for a Variety of Solvents in the Ultraviolet. *J. Opt. Soc. Am. B* **2005**, *22* (7), 1479.
6. Kedenburg, S.; Vieweg, M.; Gissibl, T.; Giessen, H. Linear Refractive Index and Absorption Measurements of Nonlinear Optical Liquids in the Visible and Near-Infrared Spectral Region. *Opt. Mater. Express* **2012**, *2* (11), 1588.
7. Auzel, F.; Baldacchini, G.; Laversenne, L.; Boulon, G. Radiation Trapping and Self-Quenching Analysis in Yb^{3+}, Er^{3+}, and Ho^{3+} Doped Y_2O_3. *Opt. Mater. (Amst).* **2003**, *24* (1–2), 103–109.
8. Püschel, S.; Kalusniak, S.; Kränkel, C.; Tanaka, H. Temperature-Dependent Radiative Lifetime of Yb:YLF: Refined Cross Sections and Potential for Laser Cooling. *Opt. Express* **2021**, *29* (7), 11106.
9. Senden, T.; Rabouw, F. T.; Meijerink, A. Photonic Effects on the Radiative Decay Rate and Luminescence Quantum Yield of Doped Nanocrystals. *ACS Nano* **2015**, *9* (2), 1801–1808.
10. Chatzigoulas, A.; Karathanou, K.; Dellis, D.; Cournia, Z. NanoCrystal: A Web-Based Crystallographic Tool for the Construction of Nanoparticles Based on Their Crystal Habit. *J. Chem. Inf. Model.* **2018**, *58* (12), 2380–2386.
11. Yu, D. C.; Rabouw, F. T.; Boon, W. Q.; Kieboom, T.; Ye, S.; Zhang, Q. Y.; Meijerink, A. Insights into the Energy Transfer Mechanism in Ce^{3+}-Yb^{3+} Codoped YAG Phosphors. *Phys. Rev. B* **2014**, *90* (16), 165126.
12. Demirbas, U.; Thesinga, J.; Kellert, M.; Kärtner, F. X.; Pergament, M. Detailed Investigation of Absorption, Emission and Gain in Yb:YLF in the 78–300 K Range. *Opt. Mater. Express* **2021**, *11* (2), 250.
13. Demirbas, U.; Thesinga, J.; Kellert, M.; Kärtner, F. X.; Pergament, M. Comparison of Different in Situ Optical Temperature Probing Techniques for Cryogenic Yb:YLF. *Opt. Mater. Express* **2020**, *10* (12), 3403.
14. Luntz-Martin, D. R.; Felsted, R. G.; Dadras, S.; Pauzauskie, P. J.; Vamivakas, A. N. Laser Refrigeration of Optically Levitated Sodium Yttrium Fluoride Nanocrystals. *Opt. Lett.* **2021**, *46* (15), 3797.
